# Integrated Single-Cell Virtual Knockout and Machine Learning Analyses Reveal a Protective Role of CKAP2 in Gastric Cancer

**DOI:** 10.3390/molecules31111901

**Published:** 2026-06-01

**Authors:** Jianhua Yang, Zheng Qiu, Wenchao Song, Xing Liu, Ting Ouyang, Jianhua Shu, Jinghui Wang, Yinfeng Yang

**Affiliations:** 1 School of Medical Informatics Engineering, Anhui University of Chinese Medicine, Hefei 230012, China; 2 Anhui Provincial Key Laboratory of Chinese Medicinal Formula, Anhui University of Chinese Medicine, Hefei 230012, China; 3 School of Integrated Chinese and Western Medicine, Anhui University of Chinese Medicine, Hefei 230012, China

**Keywords:** gastric cancer, CKAP2, virtual knockout, Mendelian randomization, machine learning, experimental validation

## Abstract

**Objective:** To elucidate the role of cytoskeleton-associated protein 2 (CKAP2) in gastric cancer (GC) progression and evaluate its prognostic and potential protective significance. **Methods:** The candidate genes for GC were identified using differential expression and weighted gene co-expression network analysis (WGCNA). A panel of machine learning algorithms was applied for obtaining the key genes and yielding the hub target gene CKAP2 through protein–protein interaction (PPI) networks and single-cell RNA sequencing analysis. Further, the functional role of CKAP2 was explored through single-cell virtual knockout and pathway enrichment analysis. Meanwhile, the survival analysis and Mendelian randomization (MR) were used to evaluate the clinical relevance and causality of CKAP2. Finally, the expression of CKAP2 was further validated in clinical tissues and GC cell lines through Western blot. **Results:** A total of 20 candidate genes were identified, of which 8 were identified as hub genes through machine learning. Integrative PPI and single-cell analyses ultimately identified CKAP2 as the key gene. Virtual knockout analysis showed that the related differentially expressed genes were significantly enriched in the 5-HT pathway. Survival analysis demonstrated that elevated CKAP2 expression was associated with improved prognosis. MR analysis further suggested that CKAP2 might act as a protective factor, while 5-HT was associated with an increased risk of GC. Experimental validation confirmed that CKAP2 is significantly upregulated in GC tissues and cell lines. **Conclusions:** CKAP2 is a potential prognostic biomarker and protective factor in GC, possibly exerting its effects through regulation of the 5-HT pathway. These findings provide new insights into the mechanisms and potential therapeutic targets of GC.

## 1. Introduction

Gastric cancer (GC) remains a major global health burden, particularly in East Asia, where it ranks among the leading causes of cancer-related mortality [1,2]. The latest data shows that there are over one million new cases each year and a large number of deaths are caused by this disease. [3]. Despite advances in early detection, surgical techniques, perioperative chemotherapy, and targeted and immunotherapies, the prognosis for patients with advanced GC remains poor [4,5]. Tumor recurrence, distant metastasis, treatment resistance, as well as obvious inter-tumor and intra-tumor heterogeneity are the main obstacles in clinical treatment [6,7]. Therefore, it is urgently necessary to identify new biomarkers and molecular regulatory factors involved in the occurrence and progression of GC and to deeply understand the mechanism of GC development.

The cell skeleton-related protein 2 (CKAP2) is a microtubule-associated protein that can regulate the cell division process and maintain genetic stability [8,9,10]. Previous studies have shown that CKAP2 is overexpressed in various cancers and is believed to be related to tumor proliferation [11,12]. However, recent studies have shown that the function of CKAP2 during mitosis may be more complex than previously thought. In some cases, it can even play a role in inhibiting tumors, especially by regulating related oncogenic signaling pathways [13,14]. In this context, the inhibitory or protective effects of CKAP2 on GC cells through unique signaling pathways have not yet been fully elucidated. Therefore, further investigation of CKAP2 may provide a novel perspective for understanding the molecular mechanisms underlying GC pathogenesis.

The latest evidence indicates that the nervous system and the signal transmission of neurotransmitters play a significant role in the occurrence of GC [15,16]. Serotonin (5-HT) is predominantly produced by enterochromaffin cells within the gastric mucosa and modulates both movement and production, and it has immediate actions on epithelial growth, preservation, and immunity [17,18]. Recent studies have demonstrated that elevated 5-HT levels and excessive activation of specific 5-HT receptors promote the progression of GC through the PI3K/AKT/mTOR signaling pathway, inhibit ferroptosis and reshape the immunosuppressive tumor microenvironment [19]. These findings suggest that serotonin plays an important role in the progression of GC.

**Figure 1:**
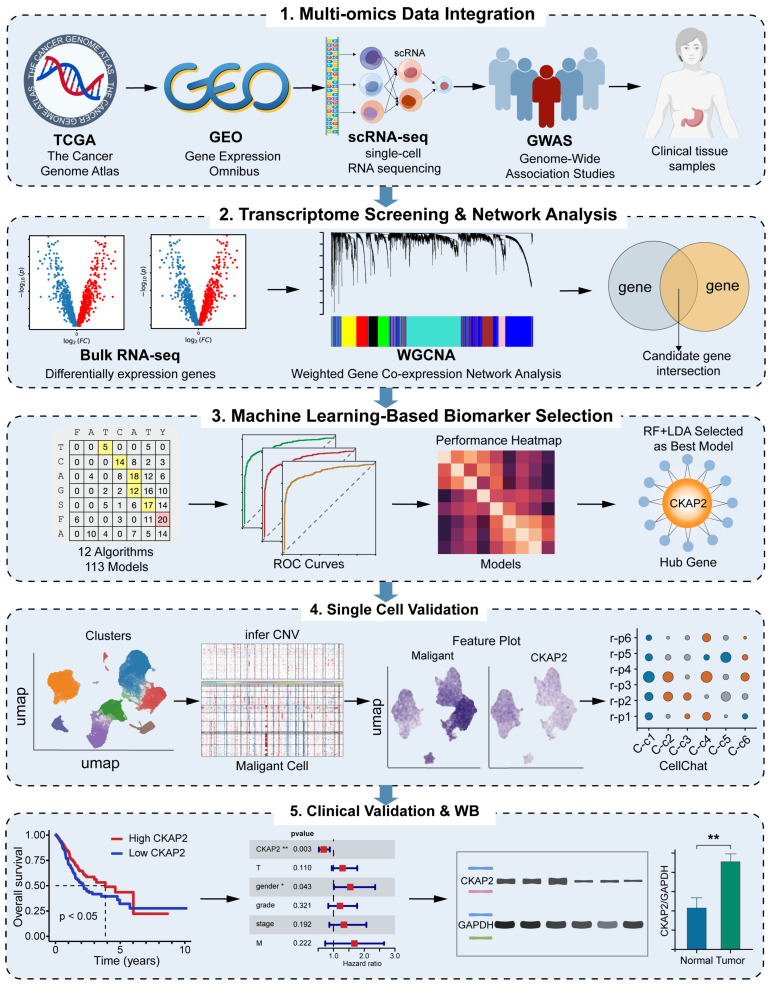
The workflow of this study. * indicates *p* value < 0.05; ** indicates *p* value < 0.01.

In this study, by integrating bulk RNA and scRNA sequencing, employing machine learning techniques, bidirectional Mendelian randomization (MR), and an integrated experimental verification framework, we elucidated the mechanism of the CKAP2 gene in GC. It was hypothesized that CKAP2 not only performs the normal functions of the cell cycle but also may affect other neural signals within the GC. Specifically, the downstream regulatory targets of CKAP2 within epithelial cell populations were precisely identified through single-cell virtual knockout (vKO) simulations. Moreover, possible causal relations between CKAP2 expression, 5-HT level, and GC development were created by bidirectional MR. Based on these results, we hypothesized that CKAP2 may exert a protective effect in GC by modulating serotonin-related pathways. Our findings provide new insights into the molecular mechanisms of GC and suggest potential targets for precision therapy. Figure 1 depicts the workflow of this study.

## 2. Materials and Methods

### 2.1. Data Collection and Preprocessing

In this study, a total of 11 datasets from the TCGA, GEO, OpenGWAS databases were collected [20,21,22,23,24,25,26,27]. Specifically, the transcriptome data and corresponding clinical pathological data of the TCGA-STAD cohort were used for differential expression analysis, WGCNA, machine learning, and immune infiltration analysis. The GSE13911 and GSE65801 datasets fulfilled dual roles, supporting both initial differential expression screening and subsequent independent validation. The GEO datasets GSE13911 and 65801 were not only used to identify differentially expressed genes, but also, together with GSE13861 and GSE29272, served as an external independent validation set for machine learning. Transcriptomic landscape was performed at single-cell level, integrating raw sequencing datasets GSE163558 and GSE184198. Additionally, GWAS summary statistics for GC, CKAP2, and 5-HT were obtained to conduct a bidirectional MR analysis, in order to evaluate the causal relationship between CKAP2-GC and 5-HT-GC progression. Details of the datasets and their sources are summarized in Table 1 and Supplementary Table S1, respectively.

### 2.2. Differential Expression Analysis

To determine the differentially expressed genes (DEGs) between the GC and control groups, raw expression matrices were log_2_-transformed and normalized to ensure cross-sample comparability. Inter-group differential analysis was conducted using the “limma” R package (version 3.62.2) [28]. For the TCGA-STAD, GSE13911, and GSE65801 datasets, DEGs were screened based on the stringent criteria of an adjusted *p*-value < 0.05 and an absolute |log_2_FC| > 0.585.

### 2.3. WGCNA for Candidate Gene Identification

Based on the transcriptome data of GC from the TCGA-STAD, a weighted gene co-expression network was constructed using the WGCNA package (version 1.73) [29]. After the optimal soft-thresholding power was determined via the pickSoftThreshold function to ensure a scale-free topology, co-expression modules were identified using the dynamic tree-cut method. Through correlation analysis between module eigengenes (MEs) and clinical phenotypes, the Meblack and Meblue modules, which exhibited the highest significance, were selected as key modules. Ultimately, within these two target modules, genes with a Kinship-based Module Eigengene (kME) greater than 0.7 were identified and defined as the candidate genes driving the progression of GC.

### 2.4. Candidate Genes Establishment Based on Machine Learning Models

To test the assessment of the robustness of the cross-platform and minimize the risk of data leakage during model development, the TCGA-STAD cohort was served as the training dataset, while another four external data sets including GSE13911, GSE65801, GSE13861, and GSE29272 were employed as an external validation set. Additionally, to get the most stable and informative diagnostic signature, 12 machine learning algorithms, i.e., Support Vector Machine (SVM), Ridge Regression (Ridge), Least Absolute Shrinkage and Selection Operator (Lasso), Partial Least Squares Generalized Linear Models (plsRglm), Random Forest (RF), Gradient Boosting Machine (GBM), Linear Discriminant Analysis (LDA), Extreme Gradient Boosting (XGBoost), Naive Bayes, Generalized Linear Model Boosting (glmBoost), Elastic Net (Enet), and Stepwise Generalized Linear Model (Stepglm), were implemented an integrative framework.

In addition, to avoid overfitting and improve prediction results, thorough hyperparameter adjustment was performed in the training set with cross-validation (CV) approaches. Specifically, we used 10-fold CV on training set to find λ that minimized classification errors for Lasso, Ridge, and Enet models. For tree-based boosting methods, in the GBM we ran a 10-fold cross validation and found the number of trees that had the lowest CV error, while a 5-fold CV was employed in XGBoost to determine the best boosting rounds alongside fixed constraints (max depth of 2 and learning rate of 1). Random Forest was constructed with a robust ensemble of 1000 trees and a minimum node size of 5. Additionally, prior to modeling, continuous variables across all cohorts were centered and scaled to eliminate scale-dependent biases. By systematically combining these algorithms for feature selection and model construction, a total of 113 different machine learning models were constructed. The final optimal model was determined by calculating the average C-index across all datasets, selecting the candidate that demonstrated the highest consistency and predictive accuracy in identifying the characteristics of GC.

### 2.5. PPI Network Construction and Hub Gene Identification

To study functional interplay between these eight candidate genes which were discovered by ML approach, a protein–protein interaction (PPI) network was constructed utilizing the STRING database [30]. We applied an intermediate interaction confidence threshold of 0.4, so as to filter the discovered protein associations. Subsequently, the generated network was visualized in the Cytospace software (version v3.10.1) and further analyzed through the CytoHubba plugin [31]. To identify the core hub genes, the network centrality was evaluated based on CytoHubba. The candidate genes were ranked according to the results of CytoHubba. Genes with top degree scores were chosen as most important hubs for the subsequent study.

### 2.6. Single-Cell Transcriptomic Analysis

In order to clarify the specific locations and expression differences in these key genes within the cells, we conducted single-cell analysis. Single-cell transcriptomic datasets were used, which included GSE163558 (consisting of 3 GC and 1 normal sample), GSE184198 (consisting of 1 GC and 1 normal sample). Those datasets were integrated and preprocessed through the “Seurat” R package [32]. Stringent quality control (QC) was first applied so as to exclude low-quality cells, more particularly when the amount of mitochondria genes is greater than 20%, or the number of detected genes falls outside of the 200–6000 range. After the QC step, there remained a total of 30,816 cells for the following work. To mitigate potential batch effects between the integrated datasets, the “Harmony” algorithm was employed, followed by log-normalization of the expression matrices. Dimensionality reduction was conducted via Principal Component Analysis (PCA) and cell subpopulations were determined through graph-based clustering with a resolution parameter set to 1.0. Finally, cell types were automatically annotated with the use of the “SingleR” package (version 2.8.0) [33].

### 2.7. Identification of Malignant Epithelial Cells via inferCNV Analysis

To distinguish malignant epithelial cells from their normal counterparts, epithelial cells were first extracted from the initial clustering results and subjected to sub-clustering analysis. Subsequently, the “inferCNV” R package (version 1.23.0) [34] was used to estimate the chromosomal copy number variations (CNVs). T cells identified within the same integrated dataset were utilized as the reference baseline to represent a diploid genomic state. The CNV signal per epithelial sub-cluster is found through comparing gene expression intensities amongst chromosome segments as compared to the reference cells. Cells with large-scale chromosomal gain or loss and high global cnv scores are malignant epithelial cells (tumor cells). On contrary, the epithelial cells maintaining relative genetic stability were classified as the non-malignant or normal epithelial cells. We prioritized these identified malignant populations for further downstream functional and investigations.

### 2.8. Virtual Knockout Simulation via scTenifoldKnk

To better understand the regulatory effects of CKAP2 on the epithelial cells, we simulated a vKO with “scTenifoldKnk”R package (version 1.0.2) [35]. First a single-cell gene regulatory network (scGRN) was built based on epithelial cells expression profile. After that, the CKAP2 gene was virtually deleted in this network by setting all its interactions as zero. The regulatory impact of this perturbation was quantified by comparing the perturbed network with the original reference network to identify differentially regulated genes (DRGs) significantly affected by the loss of CKAP2. In order to uncover which biological functions and which pathways were changed or influenced by CKAP2 we performed functional enrichment analysis with all the above obtained DRG. Statistical significance for the enrichment results was determined using an adjusted *p*-value threshold of < 0.05.

### 2.9. Cell–Cell Communication Analysis

We analyzed the signal interactions and the complex interaction relationship with different cell groups in GC microenvironment by cell-communication analysis by using the ‘CellChat’ R package [36]. Normalized gene expression data and cell type annotations derived from the scRNA-seq analysis were utilized as inputs. The database “CellChatDB. human”, which has valid ligand-receptor connections, was chosen as the standard when inferring signaling networks. The communication probability was calculated by integrating the law of mass action with the expression levels of ligands and their cognate receptors. Significantly, signaling pathways were identified via statistical permutation tests. Furthermore, cell communication was visualized through circular graphs and bubble charts to show the intensity of interactions. At the same time, the strength of input and output signals is quantified to determine the key signal types and important cell types related to GC progression.

### 2.10. Functional Enrichment and Clinical Association Analysis

To explore which biological pathways and functional relate to CKAP2 expression levels we do Gene Set Enrichment Analysis (GSEA). We stratified GC samples into high and low-CKAP2 expression groups using median value. Enrichment is done through the Gene Ontology (GO) and Kyoto Encyclopedia of Genes and Genomes (KEGG) gene set. Significant is defined by a *p*-value after adjustment that is <0.05.

To further evaluate the clinical significance of CKAP2, the association between CKAP2 and clinical pathological characteristics (including age, gender, grade, and TNM stage) was analyzed using the Wilcoxon rank sum test or the Kruskal–Wallis test. Subsequently, the prognostic impact of CKAP2 in GC was evaluated through Kaplan–Meier survival analysis. The differences in overall survival (OS) and progression-free survival (PFS) between the high-expression group and the low-expression group were assessed using the log-rank test. To determine the independent prognostic factors, we performed univariate and multivariate Cox proportional hazards regression analyses including CKAP2 expression as well as all of the other clinical covariates. Based on the independent prognostic factors identified through multivariate Cox analysis, a prognostic indicator for predicting the 1-year, 3-year, and 5-year OS probabilities of GC patients was established using the “rms” R package (version 8.0-0). The predictive accuracy of this indicator was further verified through calibration curves.

### 2.11. Immune Infiltration Profiling

Spearman rank correlation analysis was used to investigate the correlation between CKAP2 expression and tumor mutational burden (TMB). Using the CIBERSORT algorithm [37], we have determined the relative proportions of 22 infiltrating immune cell types in order to decipher the immune landscape. In addition, the ESTIMATE algorithm is used to calculate the immune score and stromal score, which reflects the overall purity of the tumor microenvironment.

### 2.12. Mendelian Randomization Analysis

The causal relationship between the hub gene and GC was evaluated using MR analysis. Given the previous analysis, we identified the interaction between CKAP2 and 5-HT, so 5-HT was also included in this analysis. Finally, a bidirectional MR model was constructed to separately assess the causal effects between CKAP2-GC and 5-HT-GC.

Instrumental variables (IVs) were selected based on genome-wide significance (*p* < 5 × 10^−6^) and stringent linkage disequilibrium thresholds (r^2^ < 0.001). Bidirectional causality was estimated primarily using the inverse variance-weighted (IVW) method (calculated via z-test). Sensitivity analyses were conducted to evaluate the reliability of the results: heterogeneity was assessed via Cochran’s Q-test (where *p* > 0.05 indicated the absence of significant heterogeneity) and horizontal pleiotropy was evaluated using the MR-Egger intercept test (utilizing *t*-test, with *p* > 0.05 suggesting no significant pleiotropic effects). Additionally, leave-one-out sensitivity analyses were performed to determine if any single SNP disproportionately influenced the causal estimates. Detailed instrumental variables and parameters are provided in Supplementary Tables S2 and S3.

### 2.13. Western Blot Validation

From the paired human GC tissues with adjacent normal parts and three major cell lines which included GES-1, MKN-45, and HGC-27 (Procell, Wuhan, China), we extracted total protein utilizing RIPA lysis buffer (Servicebio, Wuhan, China) that contained protease inhibitors. Protein concentrations were determined using a BCA assay (Servicebio, China) and samples were loaded at 20 μg per lane. Proteins were separated by 10% SDS-PAGE and subsequently transferred onto PVDF membranes (merckmillipore, Tokyo, Japan). The membranes were blocked with 5% non-fat milk at room temperature for 1 h, followed by incubation with primary antibody against CKAP2 (1:2000, 25486-1-AP, Proteintech, China) overnight at 4 °C. After washing, membranes were incubated with the secondary antibody at room temperature for 1 h. Protein bands were visualized using ECL, with GAPDH (1:10,000, 60004-1-Ig, Proteintech, Wuhan, China) serving as an internal control. Densitometric analysis was performed using ImageJ software (version 1.54r).

## 3. Results

### 3.1. Integrated Analysis Identifies Candidate Genes

**Figure 2:**
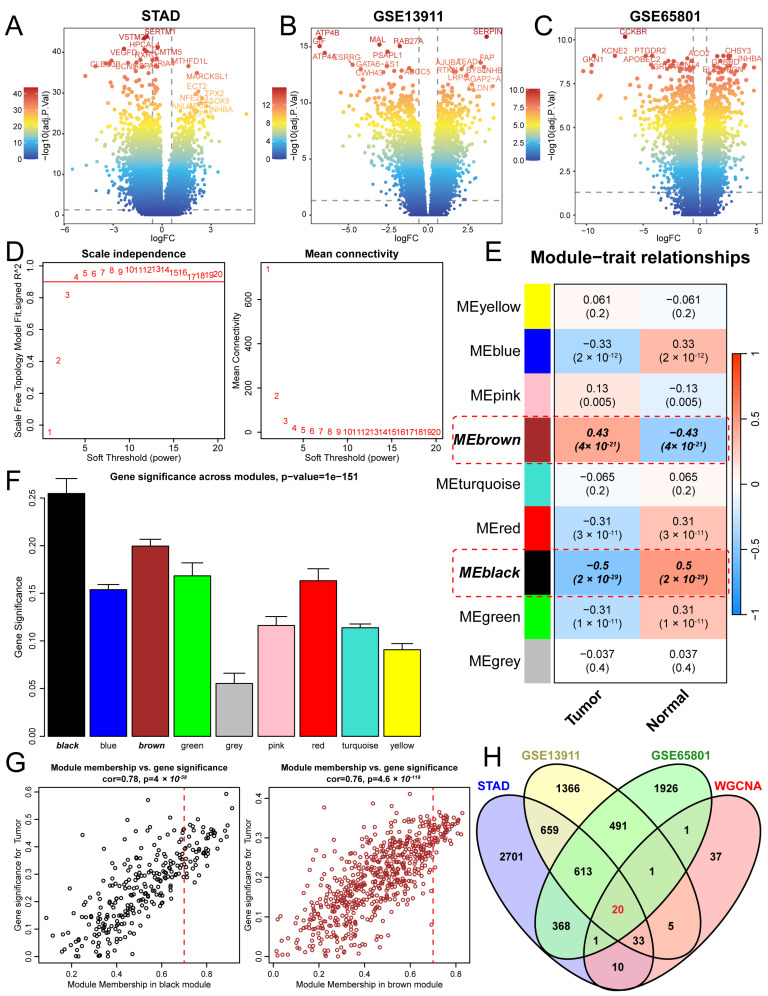
**Identification of candidate genes via integrative analysis of differential expression and WGCNA.** (**A**–**C**) Volcano plots displaying DEGs in three independent GC cohorts: STAD, GSE13911, and GSE65801. (**D**) Determination of soft-thresholding power in WGCNA, showing the scale-free topology fit index and mean connectivity across various powers. (**E**) Heatmap of module-trait relationships, highlighting modules significantly correlated with tumor and normal clinical phenotypes. (**F**) Gene significance distribution across identified co-expression modules, indicating the correlation of modules with the target phenotype. (**G**) Scatter plots showing the correlation between module membership and gene significance for the most significant modules (MEblack and MEbrown). (**H**) Venn diagram illustrating the intersection of DEGs from multiple datasets and the core modules identified by WGCNA to pinpoint key candidate genes.

To screen reliable candidate genes related to GC, we first conducted differential expression analysis in three independent transcriptome datasets. As shown in Figure 2A–C, a total of 4405 differentially expressed genes were identified in the TCGA-STAD dataset, including 2065 upregulated genes and 2340 downregulated genes. In the GSE13911 dataset, a total of 3188 differentially expressed genes were detected (1675 upregulated and 1513 downregulated). Additionally, in the GSE65801 dataset, a total of 3421 differentially expressed genes were obtained, including 1666 upregulated genes and 1755 downregulated genes.

Subsequently, we conducted a weighted gene co-expression network analysis (WGCNA) to identify gene modules with high correlations. The results showed that when the soft threshold was set at 4, the network best matched the scale-free feature (Figure 2D). Based on this, we constructed the co-expression network and analyzed the relationship between the modules and clinical phenotypes. Among all the modules, MEblack and MEbrown had the most significant correlation with the phenotype: MEblack was negatively correlated with the tumor (r = −0.50, *p* = 2 × 10^−29^), while MEbrown was positively correlated with the tumor (r = 0.43, *p* = 4 × 10^−21^) (Figure 2E). At the same time, the overall higher gene significance of these two modules further supported their importance (Figure 2F). In these two modules, we selected genes with kME > 0.7 as key genes (Figure 2G). Finally, we conducted an intersection analysis of these genes with the common differentially expressed genes in the three cohorts to obtain 20 candidate genes for subsequent machine learning analysis (Figure 2H).

### 3.2. Machine Learning Reveals Hub Genes

**Figure 3:**
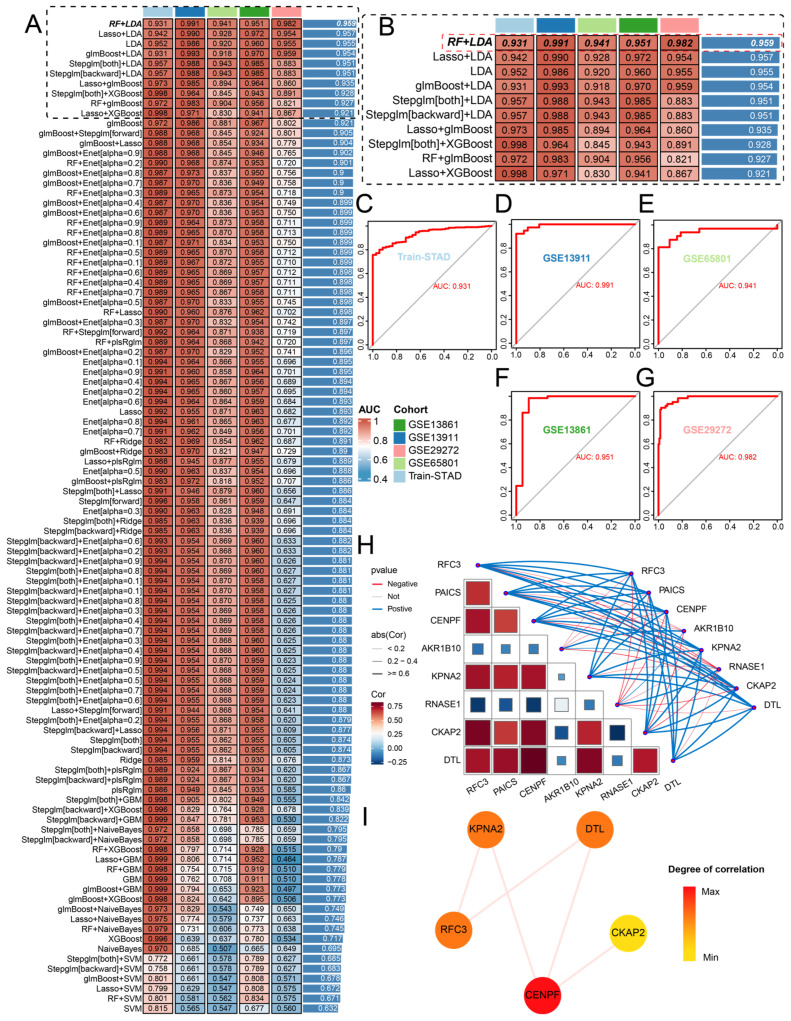
**Robust selection of machine learning models and identification of key genes.** (**A**) Heatmap illustrating the C-index for various machine learning algorithms evaluated across five independent cohorts: TCGA-STAD, GSE13911, GSE65801, GSE13861 and GSE29272. (**B**) Top 10 predictive models based on their mean C-index across the integrated datasets. (**C**–**G**) ROC curves and corresponding AUC values of the optimal machine learning model, validated across one training and four independent external datasets. (**H**) Co-expression network analysis showcasing the inter-relationships among the identified eight key signature genes derived from the optimal model. (**I**) PPI network highlighting the functional connectivity among five core hub genes.

To further screen the 20 candidate genes obtained from the above analysis, we employed machine learning. By combining 12 distinct algorithms, a total of 113 machine learning models were constructed. The TCGA-STAD dataset was utilized as the training cohort, while four independent datasets (GSE13911, GSE65801, GSE13861, and GSE29272) were employed as external validation sets. The predictive performance of these 113 models was systematically evaluated based on the C-index. Consequently, the algorithmic combination of Random Forest and Linear Discriminant Analysis “RF + LDA” exhibited the most outstanding performance, achieving the highest mean AUC of 0.959 across all integrated datasets (Figure 3A,B). The model achieved an AUC of 0.931 in the STAD training set; it also maintained excellent predictive capabilities in multiple independent external validation sets, with AUC values of 0.991, 0.941, 0.951, and 0.982, respectively (Figure 3C–G).

In addition, the best model found a main signature made up of eight significant genes, which include RFC3, PAICS, CENPF, AKR1B10, KPNA2, RNASE1, CKAP2, and DTL. To visualize the interactions among these signature genes, a co-expression network was generated using the linkET package. From Figure 3H we can see close connection among each other, particularly RFC3, PAICS, CENPF, KPNA2, CKAP2, and DTL. Furthermore, to clarify the biological interactions among these key genes, a PPI network was constructed (Figure 3I). Based on the CytoHubba assessment, the top five genes ranked by degree centrality were identified as the core hub genes (Table 2). The five key genes determined were CENPF, DTL, KPNA2, RFC3, and CKAP2.

For them to further understand the functions during GC advancement, through the GO analysis, it was revealed that these five key genes significantly enrich DNA repair and cell cycle regulatory pathways, particularly the G2/M transition (Supplementary Figure S1A). These work as one unit to move things forward with cells growing and keeping their genome. In detail, KPNA2 serves as a nuclear import vehicle for necessary controllers to get into the nucleus so RFC3 and DTL may carry out DNA replication [38]. At the same time, CKAP2 and CENPF help each other to put spindles together and divide up chromosomes [39,40]. Their robust functional interplay is required to maintain genomic stability.

Furthermore, to evaluate the prognostic correlation of these hub genes, the log-rank test was used to conduct a Kaplan–Meier survival analysis on the OS data of GC patients (Supplementary Figure S1B–E). The results of the survival analysis indicated that among the five genes, only the expression of CKAP2 showed a significant statistical association with OS (log-rank *p* = 0.024; Supplementary Figure S1E). The survival analysis of CKAP2 demonstrated that patients with high expression of CKAP2 had a significantly better prognosis than those with low expression of CKAP2 (Supplementary Figure S1E), while the other four genes (CENPF, DTL, KPNA2, and RFC3) did not show statistical significance (log-rank *p* > 0.05; Supplementary Figure S1B–E). It is noteworthy that KPNA2 was not statistically significant, but it showed a better effect compared to the other genes (CENPF, DTL, and RFC3), suggesting its potential secondary prognostic correlation (log-rank *p* = 0.073; Supplementary Figure S1D). These findings further reinforce the role of CKAP2 as a key and unique protective biomarker in the progression of GC.

### 3.3. Single-Cell Analysis Identifies CKAP2 Upregulation in Malignant Cells

**Figure 4:**
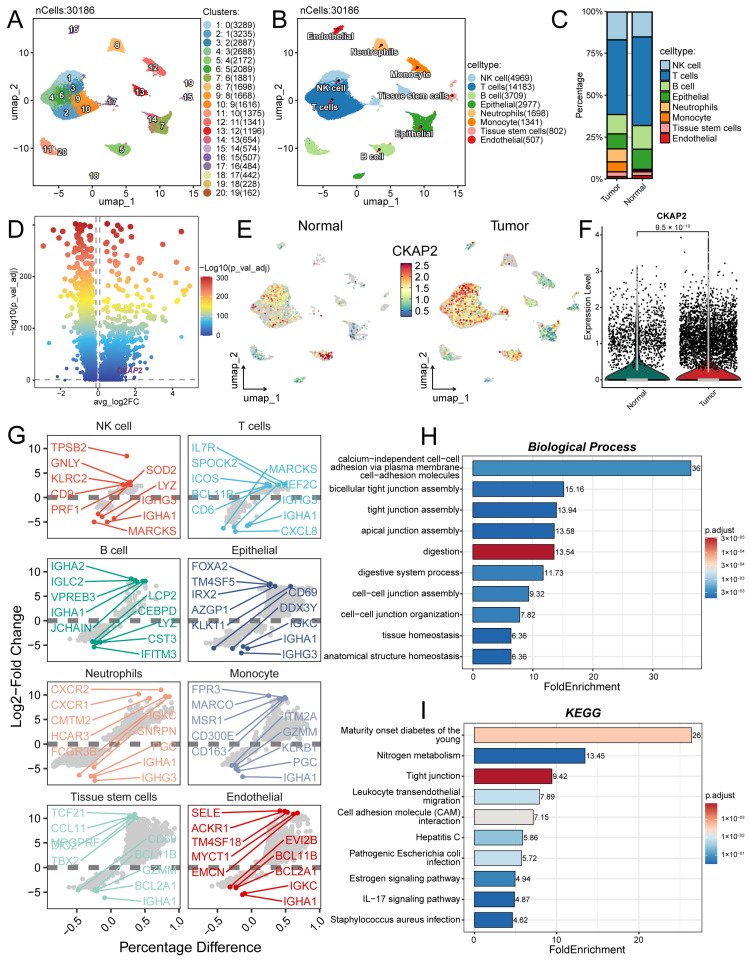
**Single-cell transcriptomic landscape and characterization of CKAP2 expression.** (**A**,**B**) UMAP visualization and cell type annotation of the single-cell dataset. (**C**) Relative proportions of identified cell lineages across groups. (**D**) Volcano plot showing global DEGs between tumor and normal samples. (**E**,**F**) Feature and violin plots illustrating the expression patterns and levels of CKAP2. (**G**) Volcano plots of differential gene expression between cell groups. (**H**,**I**) GO-BP and KEGG enrichment analysis of DEGs in the epithelial cell population.

To analyze the GC tumor microenvironment (TME) in greater detail, we conducted an analysis of the single-cell RNA sequencing (scRNA-seq) data. Firstly, we performed quality control on the data and evaluated it using indicators such as nFeature_RNA, nCount_RNA, and the ratio of mitochondria and presented the results through violin plots and scatter plots (Supplementary Figure S2A,B). Based on this, we selected highly variable genes (Supplementary Figure S2C) for subsequent dimensionality reduction and clustering analysis. After screening, a total of 30,186 high-quality cells were retained. Through unsupervised dimensionality reduction and clustering analysis, these cells were classified into 20 cell clusters (Figure 4A). Further, using SingleR for cell annotation, they were categorized into eight main cell types, including NK cells, T cells, B cells, epithelial cells, neutrophils, monocytes, tissue stem cells, and endothelial cells (Figure 4B).

The cell composition analysis revealed that the distribution of different cell types in the tumor microenvironment had undergone significant changes. Particularly, the proportion of epithelial cells in the tumor group was significantly higher than that in normal tissues (Figure 4C). In the differential analysis between the tumor and normal groups, CKAP2 showed a significant differential expression (Figure 4D) and thus was selected as the key research object for the subsequent study. The UMAP feature map indicated that the expression of CKAP2 in tumor tissues was significantly higher than that in normal tissues (Figure 4E) and this result was also confirmed consistently in the violin plot (Figure 4F). In summary, CKAP2 showed a significant upregulation trend in GC, suggesting that it may play an important role in tumor occurrence and development.

Subsequently, we conducted differential expression analysis for each of the above eight types of cells (Figure 4G). Considering that gastric adenocarcinoma originates from the malignant transformation of gastric epithelial cells [20], and that epithelial cells play a significant role in tumor progression and the remodeling of the tumor microenvironment [41], we focused on epithelial cells for the subsequent analysis. For the differentially expressed genes in epithelial cells, we further carried out GO-BP and KEGG enrichment analyses. The results showed that in addition to the common tumor-related processes, the GO-BP analysis (Figure 4H) indicated that these genes were also significantly enriched in digestive and digestive system-related functions. The KEGG analysis (Figure 4I) revealed that the estrogen signaling pathway was significantly enriched. Existing studies have shown that estrogen can regulate the serotonin system and affect its synthesis and receptor sensitivity [42]. In summary, these results suggest that the transcriptional changes in epithelial cells may be closely related to the serotonin-related signaling, thereby providing possible mechanism clues for CKAP2 participating in regulating the 5-HT signaling and affecting the progression of GC.

### 3.4. Malignant Epithelial Subtypes Reveal CKAP2-Driven Serotonergic Regulation

**Figure 5:**
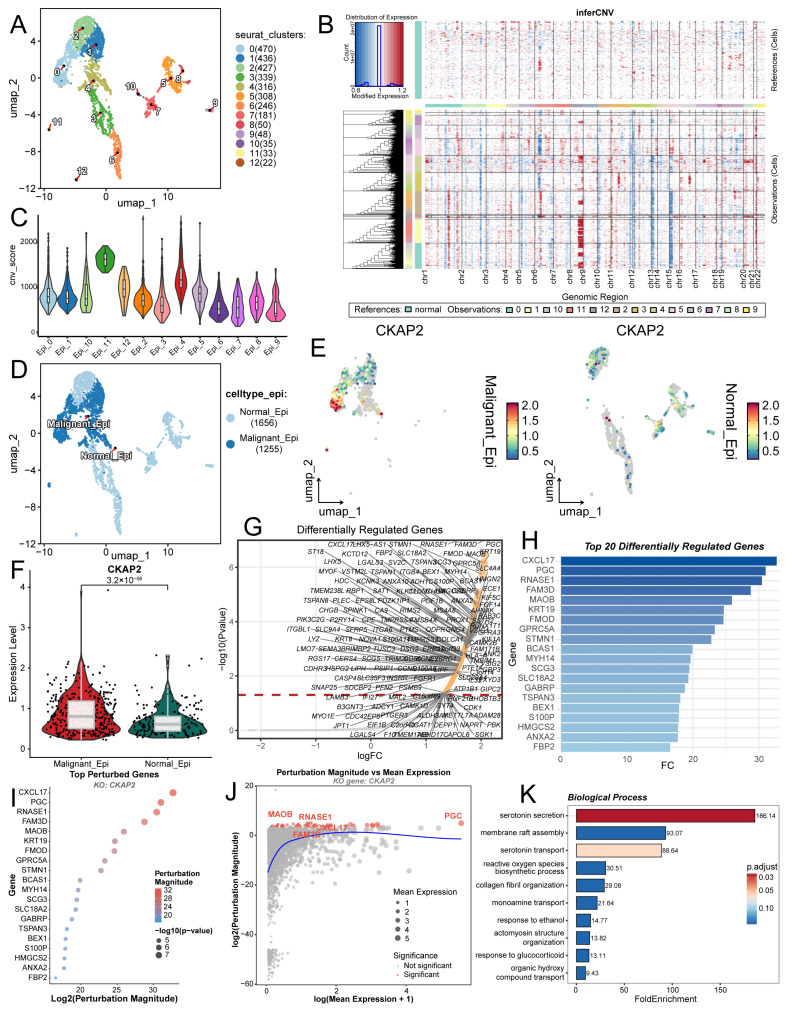
**Malignant transformation profiling and in silico perturbation analysis of CKAP2 in epithelial cells.** (**A**) UMAP visualization displaying the sub-clustering of the epithelial cell population. (**B**,**C**) CNV assessment using inferCNV, showing (**B**) the chromosomal landscape and (**C**) quantitative CNV scores across sub-clusters to identify malignant features. (**D**) Annotation of epithelial cells into malignant and normal subpopulations based on transcriptomic and CNV profiles. (**E**,**F**) Expression patterns of CKAP2 in malignant versus normal epithelial cells visualized by (**E**) feature plots and (**F**) violin plots. (**G**,**J**) In silico perturbation analysis of CKAP2. (**G**) Volcano plot highlighting significantly altered genes, (**H**,**I**) distribution of the top 20 downstream targets and (**J**) MA plot depicting the transcriptomic impact following CKAP2 virtual knockout. (**K**) Functional enrichment analysis of differentially expressed genes induced by CKAP2 perturbation.

To further analyze the heterogeneity of epithelial cells, we performed subgroup classification on them and identified a total of 13 cell clusters (Figure 5A). Subsequently, the inferCNV algorithm was used to assess the chromosomal copy number variations in each cluster, with T cells serving as the diploid reference. The results showed that some cell clusters exhibited significant genomic instability (Figure 5B,C). Based on this, Epi_0, Epi_1, Epi_4, and Epi_11 were defined as malignant epithelial cells (Malignant_Epi), while the rest were classified as normal epithelial cells (Normal_Epi) (Figure 5D). Further expression analysis indicated that CKAP2 was significantly elevated in malignant epithelial cells and this result was consistently verified in the UMAP feature map and violin plots (Figure 5E,F).

To explore the potential regulatory role of CKAP2 in epithelial cells, we conducted a vKO analysis based on the scTenifoldKnk framework. The transcriptional changes caused by the deletion of CKAP2 were presented through a volcano plot and differentially regulated genes were screened out (Figure 5G). On this basis, the 20 most significantly changed downstream genes were selected for in-depth analysis (Figure 5H,I) and the overall transcriptional changes were displayed through a MA plot (Figure 5J). Subsequently, we performed functional enrichment analysis on these 20 genes and the results showed that they were significantly enriched in the serotonin secretion-related pathways (Figure 5K). In summary, these results suggest that CKAP2 may participate in regulating serotonin-related signals in malignant epithelial cells and play a role in GC.

### 3.5. Intercellular Communication in the Tumor Microenvironment

**Figure 6:**
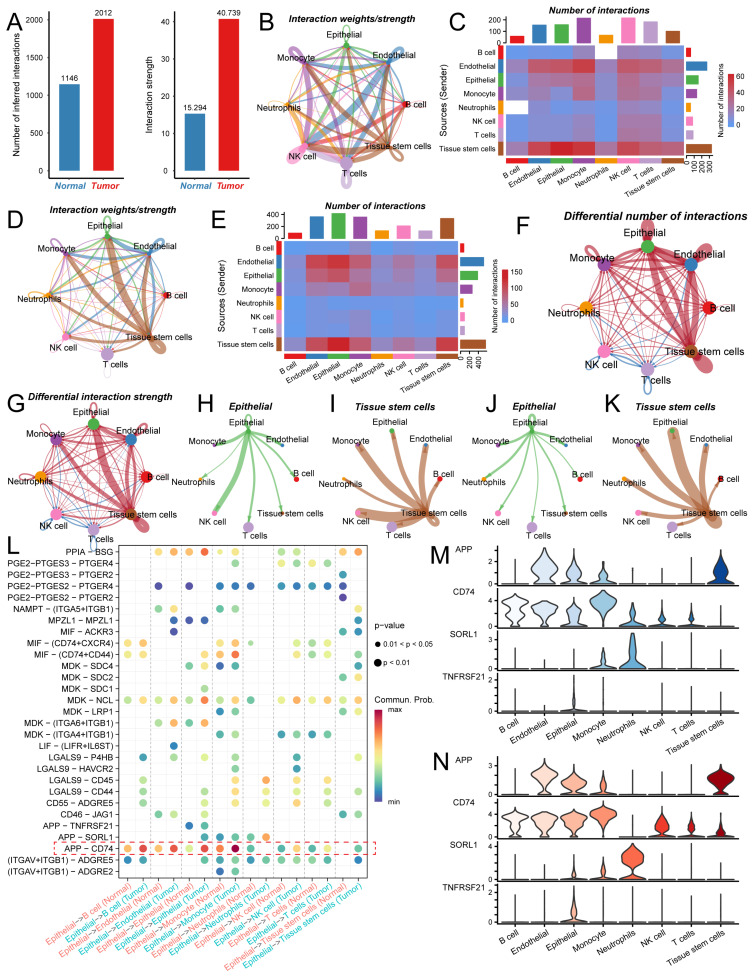
**Deciphering the altered intercellular communication landscape in the tumor microenvironment.** (**A**) The total number and strength of cellular interactions between Normal and Tumor samples. (**B**–**E**) Global cell–cell communication networks in (**B**,**C**) Normal and (**D**,**E**) Tumor tissues, visualized by network plots and heatmaps representing interaction numbers and differential strengths. (**F**,**G**) Differential interaction analysis highlighting the net changes in communication number and strength between the Tumor and Normal groups. (**H**–**K**) Detailed signaling networks of Epithelial cells and Tissue stem cells in (**H**,**I**) Normal and (**J**,**K**) Tumor samples. (**L**) Bubble plot showing the enriched ligand–receptor pairs between Epithelial cells and other cell types, comparing their interaction probabilities in Normal versus Tumor groups. (**M**,**N**) Violin plots displaying the expression levels of APP signaling pathway-related genes in (**M**) Normal and (**N**) Tumor samples.

To analyze the changes in cell-to-cell interactions during the progression of GC, we used CellChat to conduct a comparative analysis of cell communication. Firstly, we evaluated the intercellular interactions at the overall level. The results showed that the number and intensity of cell communication in tumor tissues were significantly higher than those in normal tissues (Figure 6A). This trend was further demonstrated in the network graph and heat map, suggesting that the signal transmission in the tumor microenvironment is more complex and active (Figure 6B–E). Based on this, we further conducted a differential communication analysis. The results showed that the tumor group exhibited an overall increase in the number and intensity of interactions. Among them, epithelial cells and tissue stem cells occupied a key position in the enhanced communication network (Figure 6F,G). In contrast, the cell communication in normal tissues was relatively weak, while in tumor tissues, the connections between epithelial cells and tissue stem cells, as well as between these cells and immune cells and stromal cells, were more extensive and closer (Figure 6H–K).

To further explore the changes in cell communication, we used bubble plots to display the ligand–receptor interactions between epithelial cells and other cell types (Figure 6L). Among them, the APP-CD74 signaling axis showed a higher interaction probability and significantly enhanced activity in the tumor group, with a particularly prominent difference compared to normal tissues. Based on this finding, we further analyzed the expression of APP signaling pathway-related genes, which were distributed in normal and tumor samples as shown in Figure 6M and Figure 6N, respectively. By comparing Figure 6M,N, we could observe that there were significant differences between the tumor and normal groups, especially in tumor tissues, where epithelial cells generally showed upregulation of APP-related signals. Overall, this indicates that malignant epithelial cells may participate in tumor-related signal transduction by activating the APP-CD74 signaling axis, thereby promoting the progression of GC.

### 3.6. Functional and Prognostic Significance of CKAP2

**Figure 7:**
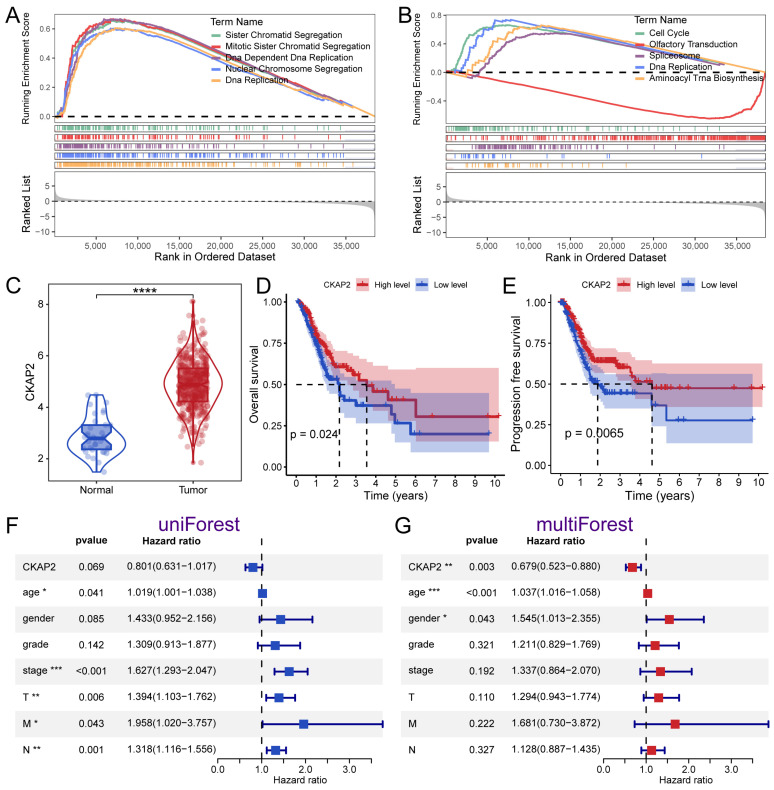
**Functional enrichment and clinical prognostic significance of CKAP2.** (**A**,**B**) GSEA revealing the (**A**) GO terms and (**B**) KEGG pathways associated with CKAP2 expression. (**C**) Expression levels of CKAP2 in Normal versus Tumor tissues. (**D**,**E**) Kaplan–Meier survival curves showing the correlation between CKAP2 expression levels and (**D**) OS as well as (**E**) PFS. (**F**,**G**) Forest plots summarizing the (**F**) univariate and (**G**) multivariate Cox regression analyses to evaluate the independent prognostic value of CKAP2 and other clinical parameters. * indicates *p* value < 0.05; ** indicates *p* value < 0.01; *** indicates *p* value < 0.001; **** indicates *p* value < 0.0001.

To explore the potential molecular mechanisms related to CKAP2, we conducted a GSEA. The GO results showed that the high expression of CKAP2 was mainly enriched in biological processes related to genomic stability and cell division, such as “sister chromatid separation”, “DNA-dependent DNA replication”, and “nuclear chromosome separation” (Figure 7A). The KEGG analysis also indicated that CKAP2 was closely related to pathways such as “cell cycle” and “DNA replication” (Figure 7B). These results suggest that CKAP2 may regulate the progression of GC by influencing the cell cycle and DNA replication processes.

As shown in Figure 7C, the results of the differential analysis indicate that the expression level of CKAP2 in GC tissues is significantly higher than that in normal tissues (adjusted *p* < 0.0001, Wilcoxon rank sum test). Further Kaplan–Meier survival analysis was performed by stratifying patients into high-expression group and low-expression group based on CKAP2 levels. The results show that high-expression group showed significantly better OS (log-rank *p* = 0.024; Figure 7D) and PFS (log-rank *p* = 0.0065; Figure 7E) than the low-expression group.

To evaluate whether CKAP2 has independent prognostic value, we conducted univariate and multivariate Cox regression analyses. The results of the univariate analysis showed that age, clinical stage, and TMN stage were significantly associated with prognosis, while CKAP2 exhibited a protective trend (Figure 7F). After further inclusion in the multivariate model and correction for other clinical variables, CKAP2 remained an independent prognostic factor (HR = 0.679, 95% CI: 0.523–0.880, *p* = 0.003, Wald test; Figure 7G).

### 3.7. Clinical Correlations of CKAP2 Expression

**Figure 8:**
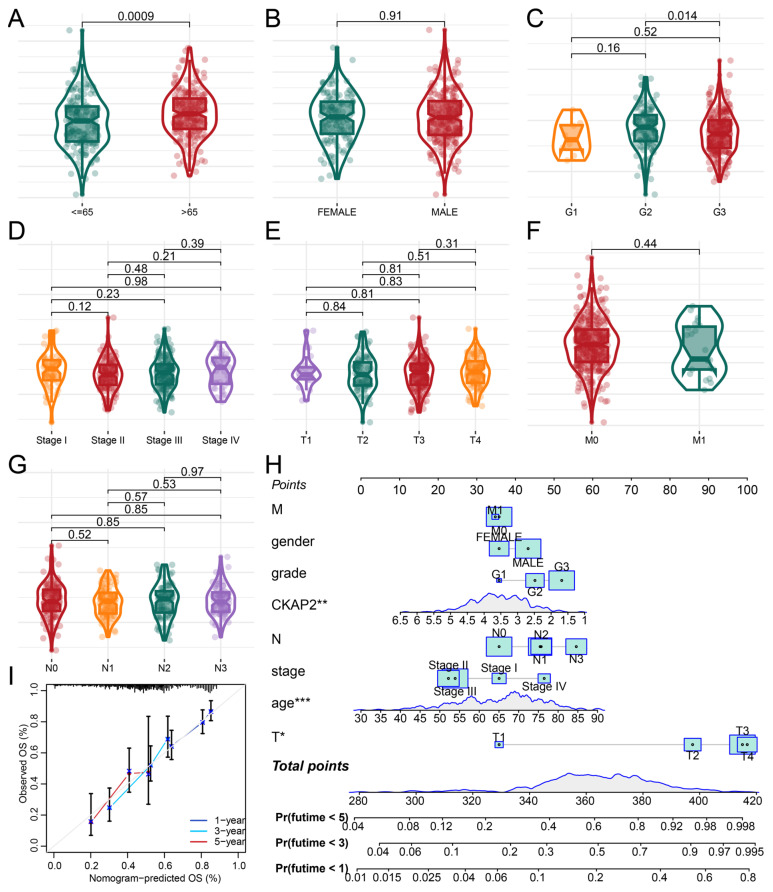
**Correlation of CKAP2 expression with clinicopathological features and construction of a prognostic nomogram.** (**A**–**G**) Association between CKAP2 expression levels and various clinicopathological characteristics in patients with GC, including (**A**) age, (**B**) gender, (**C**) histological grade, (**D**) TNM stage and (**E**–**G**) T, M and N classifications. (**H**) A prognostic nomogram integrating CKAP2 expression and independent clinical risk factors. (**I**) Calibration curves of the nomogram for predicting OS at 1, 3, and 5 years. * indicates *p* value < 0.05; ** indicates *p* value < 0.01; *** indicates *p* value < 0.001.

To explore the clinical significance of CKAP2, we compared its expression patterns under different clinical and pathological characteristics. The results showed that there were significant differences in CKAP2 expression between different age groups and histological grades. Specifically, the expression level was higher in patients over 65 years old compared to younger patients (*p* = 0.0009, Wilcoxon rank sum test; Figure 8A). Additionally, as the histological grade increased, CKAP2 expression showed an upward trend and it was significantly higher in G3 tumors than in G1 tumors (*p* = 0.014, Wilcoxon rank sum test; Figure 8C). However, there were no statistically significant differences in CKAP2 expression among gender, TNM stage, T, M and N stages (Figure 8B,D–G).

To evaluate the clinical application value of CKAP2, we combined its expression level with the independent clinical indicators selected by the multivariate Cox analysis to construct a prognostic nomogram (Figure 8H). In this model, variables such as age, gender, histological grade and TNM stage were assigned corresponding weights to calculate the individual survival probability of patients. Among them, CKAP2 contributed significantly to the model. Further, the predictive effect of the model was evaluated through calibration curves and the results showed good consistency (Figure 8I). Overall, this integrated model has good predictive performance and can provide a reference for individualized prognosis assessment of patients with GC.

### 3.8. Association with Tumor Microenvironment and Immune Infiltration

**Figure 9:**
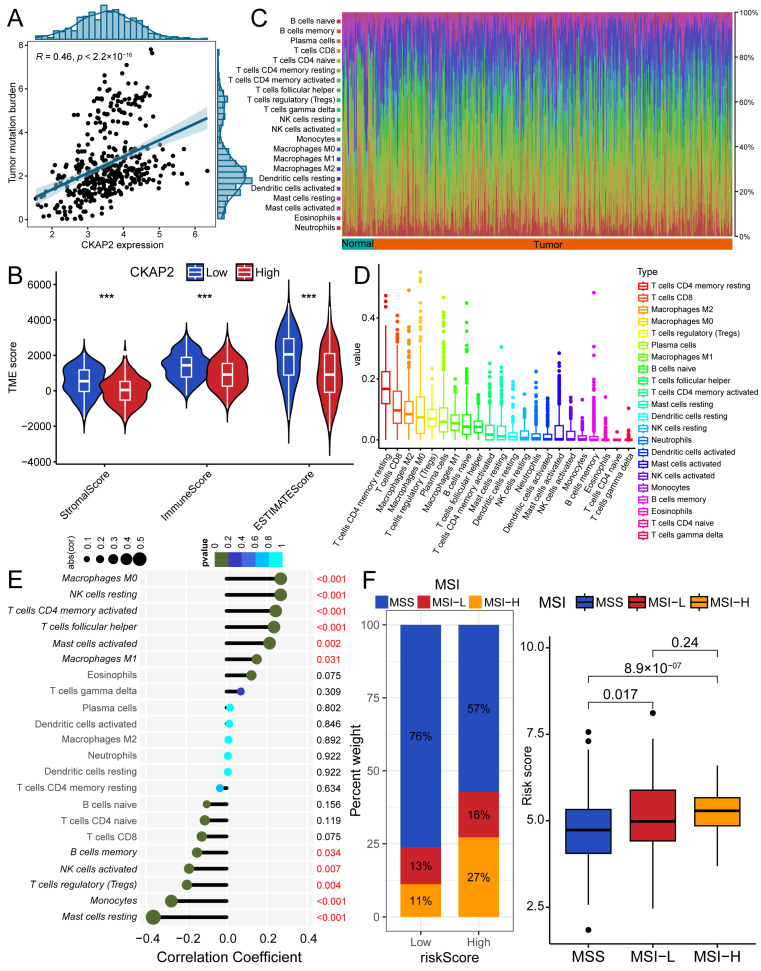
**Evaluation of the TIME and MSI.** (**A**) Correlation analysis between CKAP2 expression and TMB. (**B**) Analysis of the Tumor Microenvironment. (**C**) Proportion of Immune Cells in Each Sample. (**D**) Overall Proportion of Immune Cells. (**E**) Correlation of CKAP2 expression with specific immune cell. (**F**) Association of CKAP2 with MSI. *** indicates *p* value < 0.001.

To investigate the role of CKAP2 in the immune microenvironment of GC tumors, we analyzed the relationship between its expression and various immune-related indicators. Firstly, Spearman correlation analysis was used to assess the association between CKAP2 and tumor mutational burden (TMB). The results showed a significant positive correlation (R = 0.46, *p* < 2.2 × 10^−16^) between the two (Figure 9A), suggesting that high expression of CKAP2 may be associated with a higher load of neoantigens and genomic instability. Further, the ESTIMATE algorithm was used to evaluate the tumor microenvironment and the results indicated significant differences in immune score, stroma score, and comprehensive score between the high and low expression groups of CKAP2. Specifically, the CKAP2 high-expression group usually had a lower stroma score, suggesting a relative reduction in tumor stroma components, which may be more conducive to the infiltration of immune cells. (Figure 9B).

To understand the composition of the tumor immune microenvironment, we analyzed the infiltration of 22 types of immune cells. Figure 9C, Figure 9D, respectively, show the proportions and overall distribution of immune cells in each sample, indicating significant differences among different patients. Further, through correlation analysis, we screened the types of immune cells related to CKAP2 expression. The results showed that multiple cells were significantly correlated with it, including Macrophages M0, NK cells resting, T cells CD4 memory activated, T cells follicular helper, Monocytes and Mast cells resting (Figure 9E).

Considering that TMB is closely related to genomic stability, we further analyzed the relationship between microsatellite instability (MSI) status and the risk stratification of CKAP2. As shown in Figure 9F, the proportion of MSI-H in the high-risk group was higher (27%), while it was only 11% in the low-risk group. Additionally, the risk score of MSI-H patients was significantly higher than that of MSS patients (*p* = 8.9 × 10^−7^, Wilcoxon rank sum test). These results suggest that high CKAP2 expression is more common in the MSI-H subtype. In combination with previous studies, MSI-H typically has stronger immunogenicity and better prognosis. Therefore, the survival benefit observed in patients with high CKAP2 expression may be, to some extent, related to the characteristics of MSI-H [43].

### 3.9. Bidirectional Mendelian Randomization Analysis of CKAP2, 5-HT and GC

To further elucidate the causal relationship between CKAP2 expression and the development of GC, a bidirectional MR approach was employed to systematically dissect the potential causal network linking these factors. Given that previous in silico vKO simulations revealed that differentially regulated genes following CKAP2 knockout were significantly enriched in the 5-HT secretion pathway (Figure 5K), a bidirectional MR analysis was also conducted to investigate the causal nexus between 5-HT and GC.

As depicted in Table 3, forward MR analyses demonstrated that genetically determined CKAP2 expression was significantly associated with a decreased risk of GC (IVW OR = 0.793, 95% CI: 0.636–0.990, β = -0.231, *p* = 0.040; Supplementary Figure S3A–D). Interestingly, higher genetically predicted 5-HT levels were found to be causally linked to an increased risk of GC, exhibiting a substantial effect size (IVW OR = 5.371, 95% CI: 1.077–26.792, β = 1.681, *p* = 0.040; Supplementary Figure S4A–D). Conversely, reverse MR analyses did not identify any significant causal effect of GC status on either CKAP2 expression (*p* = 0.776) or 5-HT levels (*p* = 0.937; Supplementary Figures S3E–G and S4E–G).

To ensure the robustness of the MR estimates, comprehensive sensitivity analyses were conducted for all variables, including evaluations for horizontal pleiotropy and heterogeneity (Supplementary Tables S4 and S5). The MR-Egger intercept test showed no significant evidence of horizontal pleiotropy for the causal effects of CKAP2 (p_intercept = 0.561) or 5-HT (p_intercept = 0.314) on GC risk (Supplementary Tables S4 and S5). The Cochran’s Q test results showed that there was no significant heterogeneity among the main instrumental variables (CKAP2 on GC: Q_pval = 0.834; 5-HT on GC: Q_pval = 0.808). Additionally, the sensitivity analysis indicated that no single SNP had a dominant influence on the causal effect (Supplementary Figures S3D and S4D). Overall, these results from a genetic perspective support the protective role of CKAP2 in GC and the potential oncogenic role of 5-HT in its progression.

### 3.10. Experimental Validation of CKAP2 Expression

**Figure 10:**
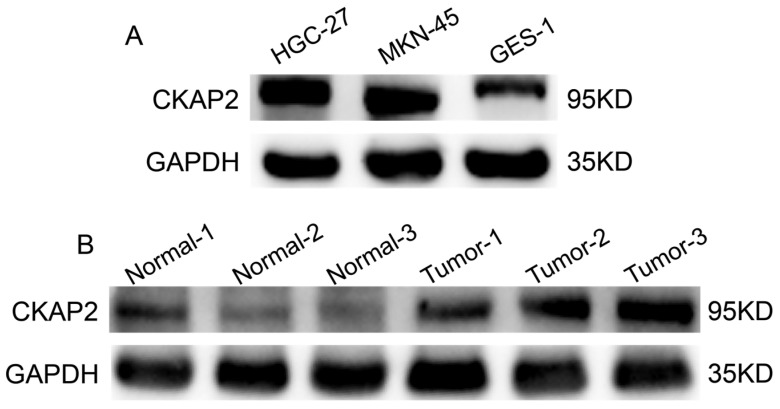
**Protein expression profiles of CKAP2 in clinical samples and gastric cancer cells.** (**A**) Western blot analysis of protein expression levels of CKAP2 in HCG-27, MKN-45 and GSE-1 cell lines. (**B**) Western blot analysis of protein expression levels of CKAP2 in normal and tumor tissue.

To verify the expression of CKAP2 in clinical samples and cells, we conducted Western blot assays on GC cell lines and tissue samples. As shown in Figure 10A, compared with the normal gastric epithelial cell line GES-1, the expression of CKAP2 protein in GC cell lines (HGC-27 and MKN-45) was significantly increased, which was consistent with the previous transcriptome analysis results. Similarly, in clinical samples, the level of CKAP2 protein in tumor tissues was also higher than that in the paired normal tissues (Figure 10B). In the experiment, GAPDH was used as the internal reference to ensure the consistency of the sample loading volume for each sample. Overall, these results further confirmed the upregulated expression of CKAP2 in GC at the protein level.

## 4. Discussion

In the present study, an integrated multi-omics framework combined with causal inference was employed to systematically elucidate the functional role and clinical significance of CKAP2 in GC progression. Through the integration of bulk transcriptomics, single-cell RNA sequencing, machine learning-based feature selection, virtual knockout simulations and bidirectional Mendelian randomization, our findings suggest that CKAP2 suppresses tumor progression by antagonizing the serotonin signaling axis.

This regulatory nexus is substantiated by evidenced from both transcriptomic simulations and genetic causal inference. Specifically, single-cell virtual knockout analysis demonstrated that loss of CKAP2 significantly perturbed genes enriched in serotonin secretion pathways (Figure 5K). This mechanism was further substantiated by bidirectional MR analysis, through which it was demonstrated that the risk of GC is increased by elevated 5-HT levels, while a protective effect is exerted by higher expression of CKAP2 (Supplementary Figures S3 and S4). These findings strengthen the biological plausibility that CKAP2 restrains GC progression, through suppression of serotonergic signaling. Previous studies have shown that serotonin promotes gastrointestinal tumor growth by enhancing proliferation signaling, metabolic adaptation and immunosuppressive remodeling [44,45,46]. Our data place CKAP2 upstream of this pathway and identify a previously underrecognized intracellular brake on serotonin-driven malignancy. This finding may be of translational relevance because several serotonergic drugs are already clinically available [47,48].

The expression landscape of CKAP2 in GC was first characterized. It was observed that CKAP2 is significantly overexpressed in GC tissues and malignant epithelial cells at both the transcriptomic and protein levels, as validated by Western blot analysis in cell lines and tumor tissues (Figure 10A,B). This observation is consistent with prior studies reporting CKAP2 upregulation in various malignancies, where it typically functions as an oncogenic driver promoting mitosis [9,12,49,50,51]. However, markedly improved OS and PFS were observed in GC patients with elevated CKAP2 expression (Figure 7D,E). Its role as a significant independent protective factor was further established by multivariate Cox regression analysis (Figure 7F,G). These findings suggest that CKAP2 in GC may not only play a traditional role in cell cycle regulation but may also have certain anti-tumor effects [52,53]. This effect may be related to its regulation of 5-HT related pathways, such as by influencing the release of 5-HT, thereby changing the metabolic state of the tumor microenvironment to some extent and thereby limiting tumor progression.

Another phenomenon worth noting is that although the high expression of CKAP2 is mainly enriched in proliferation-promoting pathways such as the cell cycle and DNA replication, clinical survival analysis shows that its high expression is significantly correlated with better prognosis in GC patients (Figure 7A–E). Further analysis reveals that the expression of CKAP2 is negatively correlated with the infiltration of immunosuppressive Tregs, suggesting that it may be associated with a more favorable immune state (Figure 9E). At the same time, the expression of CKAP2 is positively correlated with TMB and is more common in the immunogenic MSI-H subtype [54,55] (Figure 9A,F). Overall, CKAP2 may jointly affect prognosis through multiple mechanisms: on one hand, it interacts with the 5-HT-related pathway, on the other hand, it is associated with a more active immune environment, accompanied by a lower level of Tregs infiltration. These factors may jointly promote the anti-tumor immune response, thereby leading to better clinical outcomes.

Single-cell analysis provides a more detailed perspective for understanding the role of CKAP2. Its significant upregulation in malignant epithelial cells suggests that the effect of CKAP2 may originate from the tumor cells themselves rather than being merely a result of interference from the matrix components (Figure 5E,F). At the same time, CellChat analysis revealed that the cell communication centered around epithelial cells in tumor tissues was significantly enhanced, with the APP–CD74 signaling axis being particularly prominent (Figure 6L–N). Consistent with emerging paradigms in tumor biology [56,57,58], our observations indicate that CKAP2 drives GC progression by integrating intracellular transcriptional reprogramming with the modulation of epithelial–immune crosstalk. Thus, CKAP2 may function at the interface of tumor cell biology and the microenvironment.

Despite the integration of in silico single-cell simulations and Mendelian randomization analyses, certain limitations must be acknowledged. Specifically, the exact molecular pathways underlying the regulation of 5-HT by CKAP2 have yet to be functionally validated through in vivo and in vitro experiments. Future investigations should be directed toward experimental approaches, such as the targeted knockdown or overexpression of CKAP2, to acquire direct biochemical evidence of its crosstalk with 5-HT receptors.

In summary, CKAP2 is redefined by the present study from a conventional proliferation marker into a protective regulator, whereby the serotonin-mediated oncogenic axis in GC is effectively disrupted. A novel mechanistic framework is provided by functionally linking CKAP2 to the inhibition of the serotonergic axis. CKAP2 is proposed as a promising prognostic biomarker that targets the 5-HT signaling axis to suppress GC progression.

## 5. Conclusions

In this study, a comprehensive analysis integrating transcriptomic data, single-cell virtual knockout, machine learning, and experimental validation was performed to systematically investigate the role of CKAP2 in GC. Our results demonstrate that high expression of CKAP2 is significantly associated with favorable clinical outcomes and may serve as an independent protective factor in GC.

Mechanistically, our findings suggest that CKAP2 may suppress malignant progression by modulating 5-HT-related signaling pathways. This hypothesis is supported by single-cell virtual knockout analysis and bidirectional Mendelian randomization results, which indicated a causal relationship between CKAP2, 5-HT, and GC risk. In addition, CKAP2 expression was closely associated with tumor immune features, particularly showing a negative correlation with Treg infiltration, implying a potential role in shaping the anti-tumor immune microenvironment.

Overall, this study highlights the functional and clinical significance of CKAP2 in GC and proposes the CKAP2/5-HT axis as a promising biomarker and therapeutic target. These findings provide a foundation for future mechanistic studies and support the development of personalized treatment strategies for GC.

## Figures and Tables

**Table 1:** The detailed information of the datasets used in this study.

Datasets Name	Data Type	Sample Number	Sources
TCGA-STAD	bulk RNA sequencing data	412 GC tissues and 36 normal GC tissues.	TCGA [20]
Clinical TCGA-STAD	Clinical STAD data	432 patients with STAD	TCGA [20]
GSE13911	bulk RNA sequencing data	38 GC specimens and 31 controls.	GEO [21]
GSE65801	bulk RNA sequencing data	32 GC samples and 32 normal tissues	GEO [22]
GSE13861	bulk RNA sequencing data	65 GC samples and 19 normal tissues	GEO [23]
GSE29272	bulk RNA sequencing data	62 GC samples and 134 normal tissues	GEO [24]
GSE163558	single-cell RNA data	3 tumor tissues and 1 normal tissue.	GEO [25]
GSE184198	single-cell RNA data	1 tumor tissues and 1 normal tissue.	GEO [26]
GWAS-GC	GWAS statistics data	GWAS on GC in 16,380,305 nsnp	GWAS [27]
GWAS-CKAP2	pQTL statistics data	GWAS on CKAP2 in 10,534,735 nsnp	GWAS [27]
GWAS-5-HT	pQTL statistics data	GWAS on 5-HT in 2,545,835 nsnp	GWAS [27]

pQTL: Protein quantitative trait locus; STAD: stomach adenocarcinoma.

**Table 2:** Network topology parameters of the eight crucial genes.

No	Crucial Gene	Degree	MCC	MNC	EPC
1	*CENPF*	3	3	1	2.953
2	*DTL*	2	2	1	2.723
3	*KPNA2*	2	2	1	2.709
4	*RFC3*	2	2	1	2.69
5	*CKAP2*	1	1	1	2.141
6	*PAICS*	/	/	/	/
7	*AKR1B10*	/	/	/	/
8	*RNASE1*	/	/	/	/

**Table 3:** CKAP2 and its association with 5-HT in the MR analyses.

Exposure	Outcome	No. of SNPs	Methods	OR (95% CI)	β (SE)	*p*
**The forward MR analysis**						
CKAP2	GC	9	IVW	0.793 (0.636–0.990)	−0.231 (0.113)	0.040
5-HT	GC	10	IVW	5.371 (1.077–26.792)	1.681 (0.820)	0.040
**The reverse MR analysis**						
GC	CKAP2	9	IVW	1.013 (0.926–1.109)	0.013 (0.046)	0.776
GC	5-HT	4	IVW	0.999 (0.980–1.018)	−0.001 (0.010)	0.937

## Data Availability

Publicly available datasets were analyzed in this study, and the detailed data sources and accession information are provided in Section 2.1.

## Supplementary Materials

The following supporting information can be downloaded at: https://www.mdpi.com/article/10.3390/molecules31111901/s1, Figure S1: Differential expression analysis of a single gene. (A–E) The mRNA expression levels of ASPM, BRCA1, CCNA2, CHEK1 and KIF2C in normal and GC tissue; Figure S2: (A–D) The forward MR analysis on the casual effect of BRCA1 for GC. (A) The scatter plot of the association between BRCA1 and GC. (B) The forest plot was used to show the MR estimate and 95% CI value (black line segment) for each SNP. (C) The funnel plot of heterogeneity analysis. (D) The forest plot of pleiotropy analysis. (E–H) The reverse MR analysis for GC on BRCA1. (E) The scatter plot of the association between GC and BRCA1. (F) The forest plot was used to show the MR estimate and 95% CI value (black line segment) for each SNP. (G) The funnel plot of heterogeneity analysis. (H) The forest plot of pleiotropy analysis. Figure S3: (A–D) The forward MR analysis: casual effect of NADPH on GC. (A) The scatter plot of the association between NADPH and GC. (B) The forest plot was used to show the MR estimate and 95% CI value (black line segment) for each SNP. (C) The funnel plot of heterogeneity analysis. (D) The forest plot of pleiotropy analysis. (E–H) The reverse MR analysis for GC on NADPH. (E) The scatter plot of the association between GC and NADPH. (F) The forest plot was used to show the MR estimate and 95% CI value (black line segment) for each SNP. (G) The funnel plot of heterogeneity analysis. (H) The forest plot of pleiotropy analysis. Figure S4: (A–D) The forward MR analysis: casual effect of NADH on GC. (A) The scatter plot of the association between NADH and GC. (B) The forest plot was used to show the MR estimate and 95% CI value (black line segment) for each SNP. (C) The funnel plot of heterogeneity analysis. (D) The forest plot of pleiotropy analysis. (E–H) The reverse MR analysis for GC on NADH. (E) The scatter plot of the association between GC and NADH. (F) The forest plot was used to show the MR estimate and 95% CI value (black line segment) for each SNP. (G) The funnel plot of heterogeneity analysis. (H) The forest plot of pleiotropy analysis. Figure S5: (A–D) The forward MR analysis: casual effect of BRCA1 on NADPH. (A) The scatter plot of the association between BRCA1and NADPH. (B) The forest plot was used to show the MR estimate and 95% CI value (black line segment) for each SNP. (C) The funnel plot of heterogeneity analysis. (D) The forest plot of pleiotropy analysis. (E–H) The reverse MR analysis for NADPH on BRCA1. (E) The scatter plot of the association between NADPH and BRCA1. (F) The forest plot was used to show the MR estimate and 95% CI value (black line segment) for each SNP. (G) The funnel plot of heterogeneity analysis. (H) The forest plot of pleiotropy analysis. Figure S6: (A–D) The forward MR analysis: casual effect of BRCA1 on NADH. (A) The scatter plot of the association between BRCA1and NADH. (B) The forest plot was used to show the MR estimate and 95% CI value (black line segment) for each SNP. (C) The funnel plot of heterogeneity analysis. (D) The forest plot of pleiotropy analysis. (E–H) The reverse MR analysis for NADH on BRCA1. (E) The scatter plot of the association between NADH and BRCA1. (F) The forest plot was used to show the MR estimate and 95% CI value (black line segment) for each SNP. (G) The funnel plot of heterogeneity analysis. (H) The forest plot of pleiotropy analysis. Figure S7: (A–D) The forward MR analysis: casual effect of NADH on NADPH. (A) The scatter plot of the association between NADH and NADPH. (B) The forest plot was used to show the MR estimate and 95% CI value (black line segment) for each SNP. (C) The funnel plot of heterogeneity analysis. (D) The forest plot of pleiotropy analysis. (E-H) The reverse MR analysis for NADPH on NADH. (E) The scatter plot of the association between NADPH and NADH. (F) The forest plot was used to show the MR estimate and 95% CI value (black line segment) for each SNP. (G) The funnel plot of heterogeneity analysis. (H) The forest plot of pleiotropy analysis. Table S1: The data sources of the datasets used in this study; Table S2: The Filter Criteria of SNPs. Table S3: The Instrumental Variables. Table S4: The Instrumental Variables. Table S5: Pleiotropy and heterogeneity analysis between BRCA1 and GC. Table S6: Pleiotropy and heterogeneity analyses between NADPH and GC. Table S7: Pleiotropy and heterogeneity analysis between NADH and GC. Table S8: Pleiotropy and heterogeneity analyses between BRCA1 and NADPH. Table S9: Pleiotropy and heterogeneity analyses between BRCA1 and NADH. Table S10: Pleiotropy and heterogeneity analyses between NADH and NADPH.
