# Supplementary material for: Integrated Single-Cell Virtual Knockout and Machine Learning Analyses Reveal a Protective Role of CKAP2 in Gastric Cancer
Source: Molecules. 2026 Jun 1;31(11):1901. doi: 10.3390/molecules31111901 (PMC13257747; doi:10.3390/molecules31111901)
Supplement: Supplementary file 1 [file molecules-31-01901-s001.zip › molecules-4173226-supplementary.pdf]

Integrated Single-Cell Virtual Knockout and Machine Learning Analyses Reveal a Protective Role of CKAP2 in Gastric Cancer

Jianhua Yang [1], Zheng Qiu [1], Wenchao Song [1], Xing Liu [1], Ting Ouyang [1], Jinghui Wang[2,3,*] and Yinfeng Yang[1,2,*]

1. School of Medical Informatics Engineering, Anhui University of Chinese Medicine, Hefei, Anhui 230012, China
2. Anhui Provincial Key Laboratory of Chinese Medicinal Formula, Anhui University of Chinese Medicine, Hefei, China
3. School of Integrated Chinese and Western Medicine, Anhui University of Chinese Medicine, Hefei, Anhui 230012, China

* Correspondence: jhwang_dlut@163.com (J.W.); yinfengyang@yeah.net (Y.Y.)

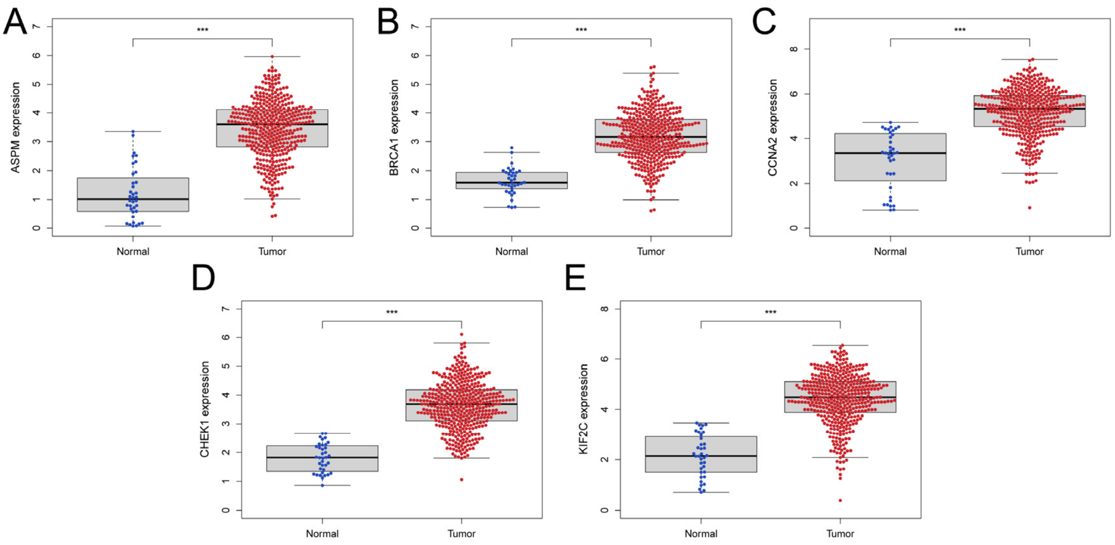

Supplementary Figure S1. Differential expression analysis of a single gene. (A-E) The mRNA expression levels of ASPM, BRCA1, CCNA2, CHEK1 and KIF2C in normal and GC tissue.

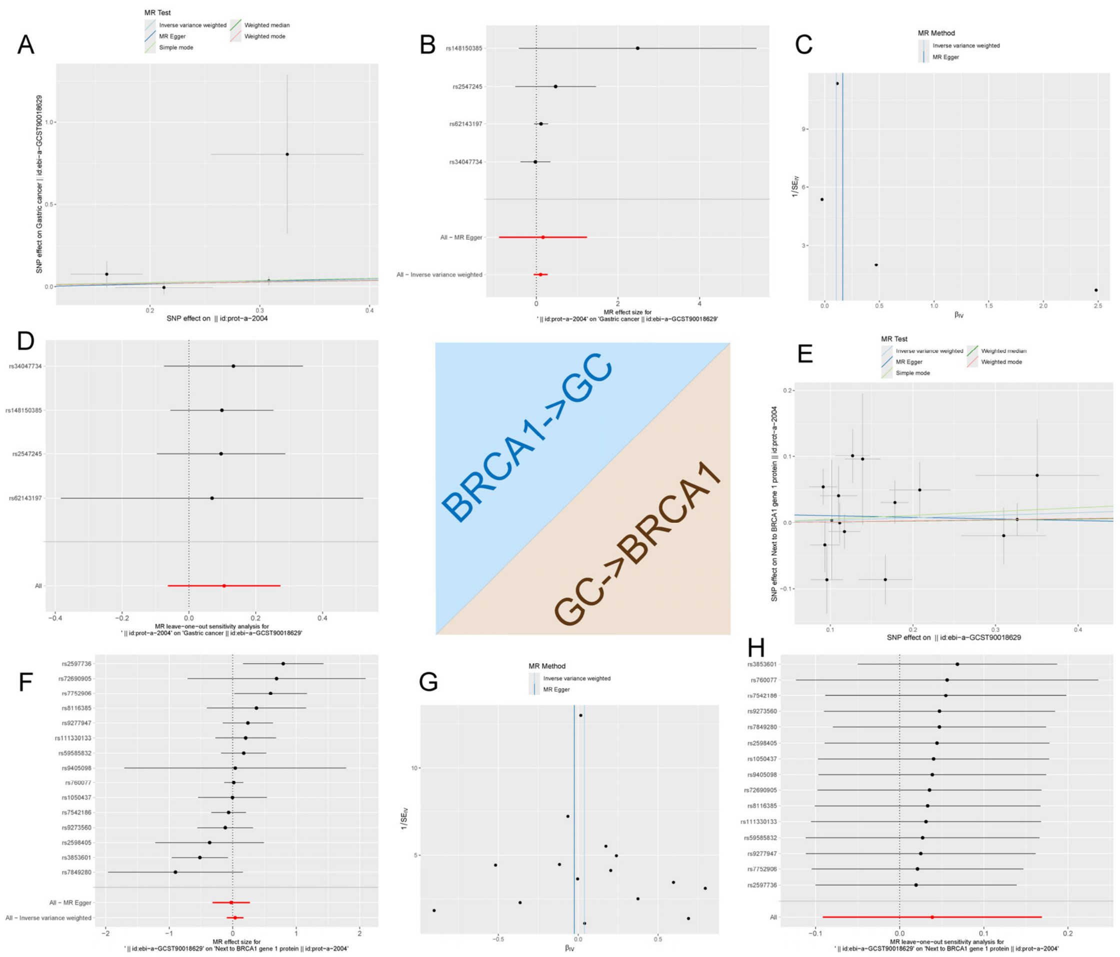

Supplementary Figure S2. (A-D) The forward MR analysis on the casual effect of BRCA1 for GC. (A) The scatter plot of the association between BRCA1 and GC. (B) The forest plot was used to show the MR estimate and 95% CI value (black line segment) for each SNP. (C) The funnel plot of heterogeneity analysis. (D) The forest plot of pleiotropy analysis. (E-H) The reverse MR analysis for GC on BRCA1. (E) The scatter plot of the association between GC and BRCA1. (F) The forest plot was used to show the MR estimate and 95% CI value (black line segment) for each SNP. (G) The funnel plot of heterogeneity analysis. (H) The forest plot of pleiotropy analysis.

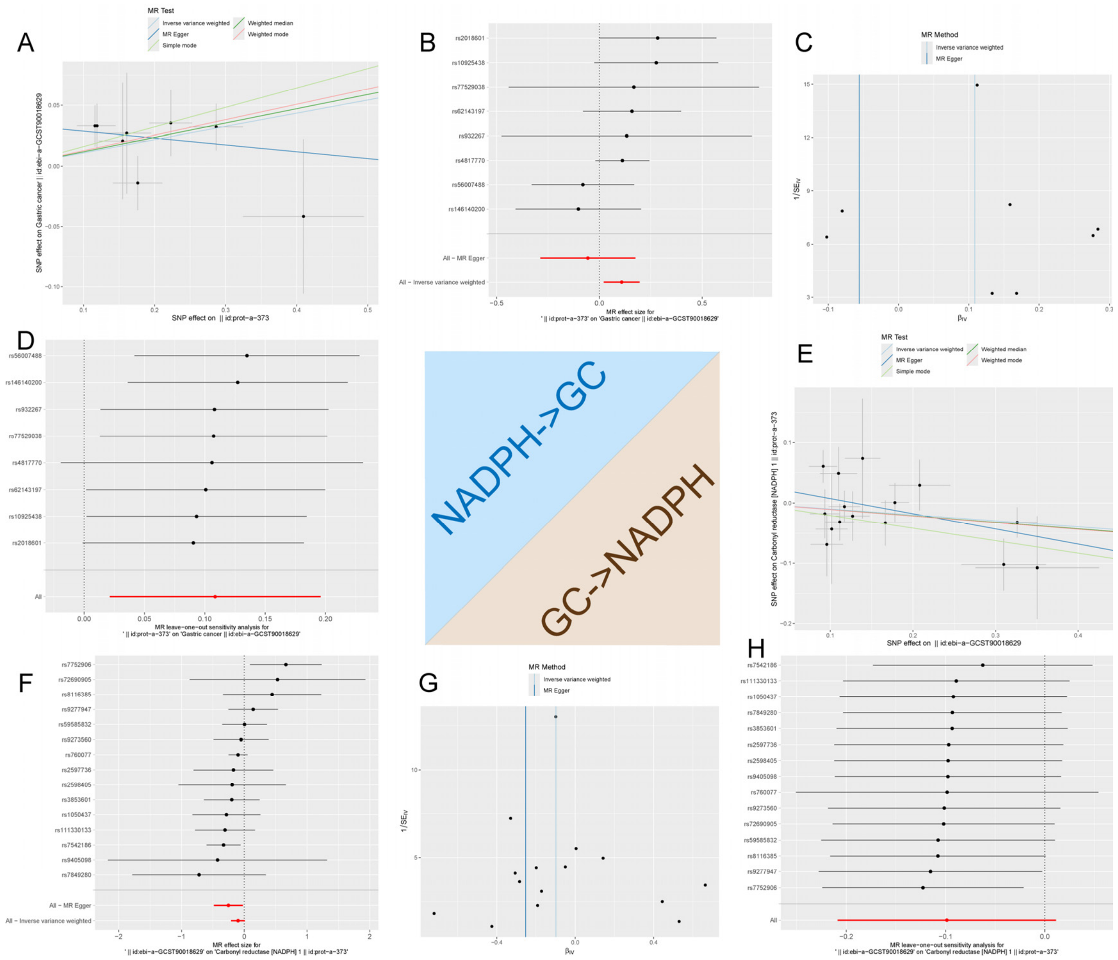

Supplementary Figure S3. (A-D)The forward MR analysis: casual effect of NADPH on GC. (A) The scatter plot of the association between NADPH and GC. (B) The forest plot was used to show the MR estimate and 95% CI value (black line segment) for each SNP. (C) The funnel plot of heterogeneity analysis. (D) The forest plot of pleiotropy analysis. (E-H) The reverse MR analysis for GC on NADPH. (E) The scatter plot of the association between GC and NADPH. (F) The forest plot was used to show the MR estimate and 95% CI value (black line segment) for each SNP. (G) The funnel plot of heterogeneity analysis. (H) The forest plot of pleiotropy analysis.

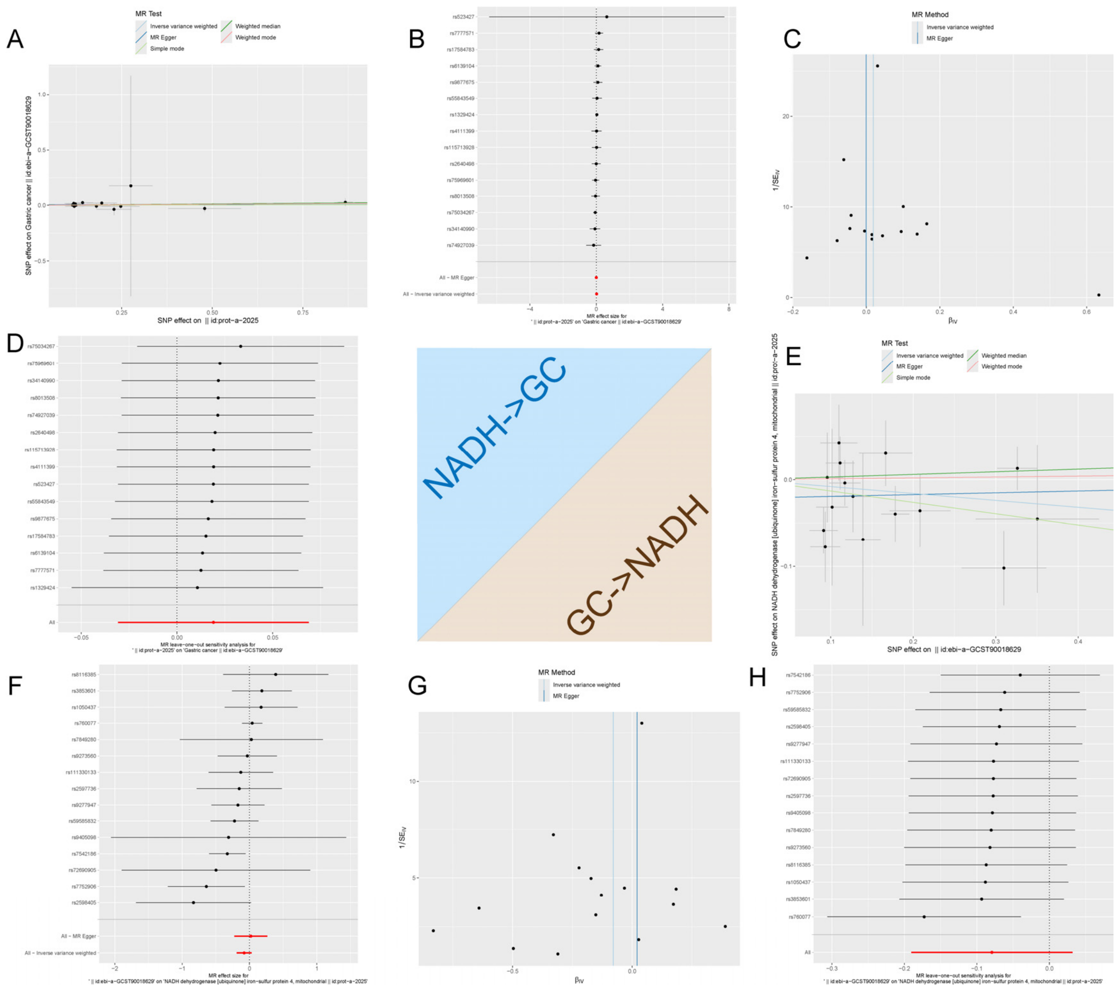

Supplementary Figure S4. (A-D)The forward MR analysis: casual effect of NADH on GC. (A) The scatter plot of the association between NADH and GC. (B) The forest plot was used to show the MR estimate and 95% CI value (black line segment) for each SNP. (C) The funnel plot of heterogeneity analysis. (D) The forest plot of pleiotropy analysis. (E-H) The reverse MR analysis for GC on NADH. (E) The scatter plot of the association between GC and NADH. (F) The forest plot was used to show the MR estimate and 95% CI value (black line segment) for each SNP. (G) The funnel plot of heterogeneity analysis. (H) The forest plot of pleiotropy analysis.

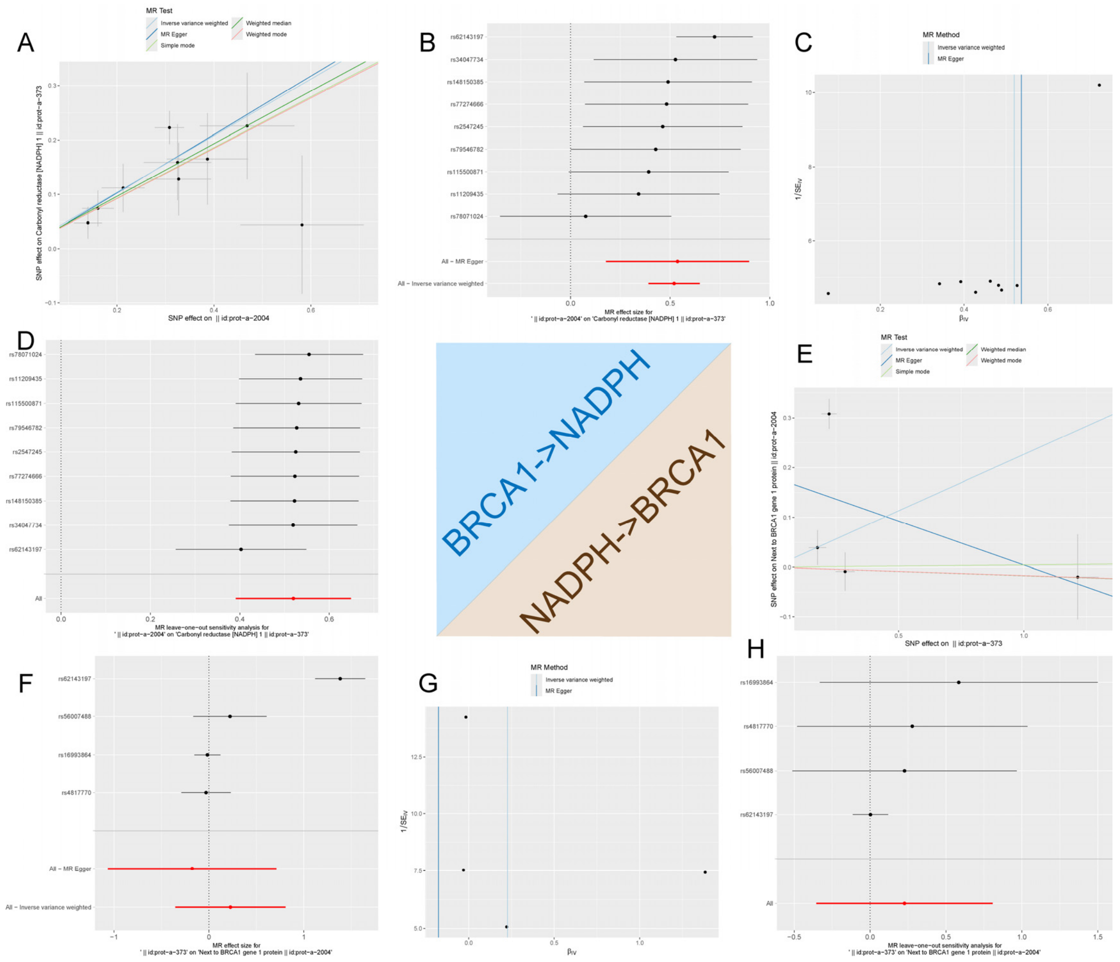

Supplementary Figure S5. (A-D)The forward MR analysis: casual effect of BRCA1 on NADPH. (A) The scatter plot of the association between BRCA1and NADPH. (B) The forest plot was used to show the MR estimate and 95% CI value (black line segment) for each SNP. (C) The funnel plot of heterogeneity analysis. (D) The forest plot of pleiotropy analysis. (E-H) The reverse MR analysis for NADPH on BRCA1. (E) The scatter plot of the association between NADPH and BRCA1. (F) The forest plot was used to show the MR estimate and 95% CI value (black line segment) for each SNP. (G) The funnel plot of heterogeneity analysis. (H) The forest plot of pleiotropy analysis.

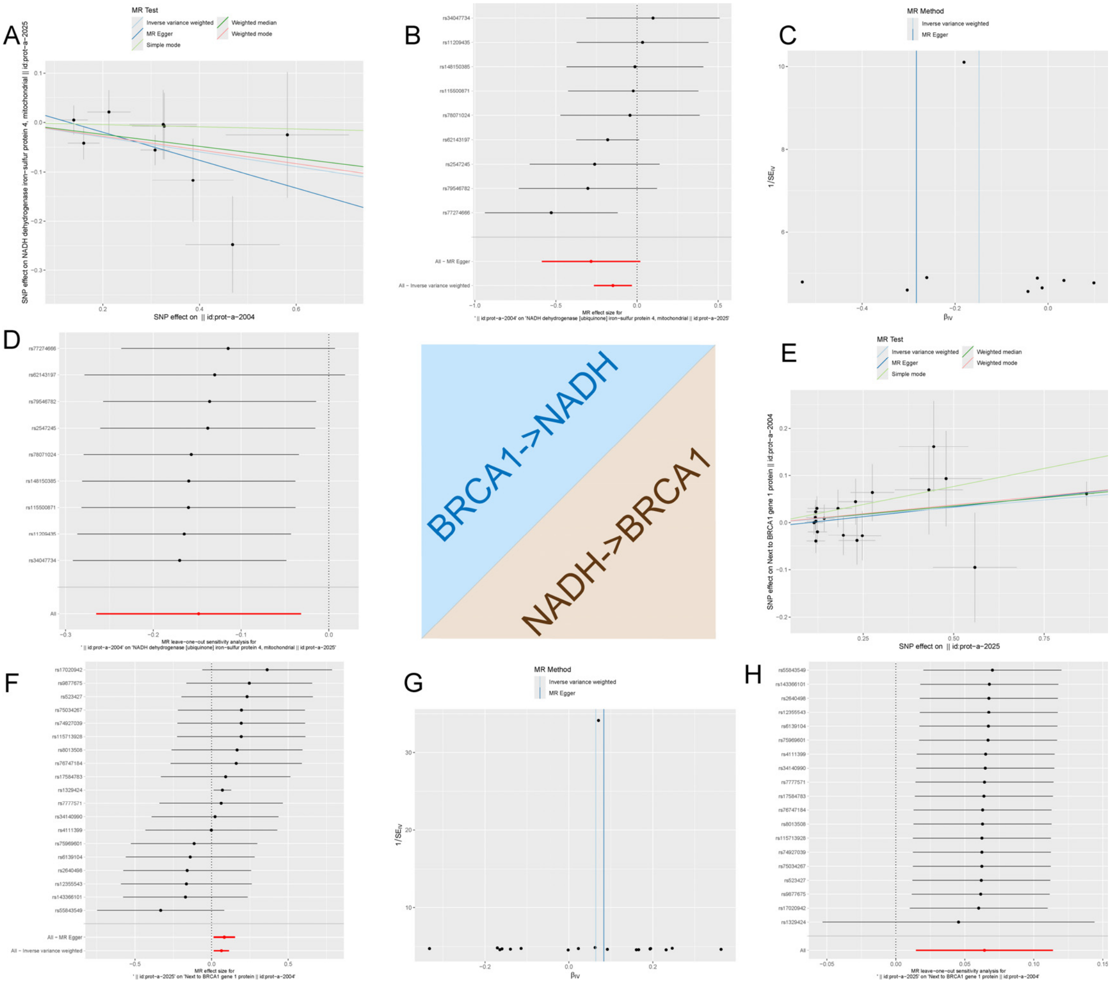

Supplementary Figure S6. (A-D)The forward MR analysis: casual effect of BRCA1 on NADH. (A) The scatter plot of the association between BRCA1and NADH. (B) The forest plot was used to show the MR estimate and 95% CI value (black line segment) for each SNP. (C) The funnel plot of heterogeneity analysis. (D) The forest plot of pleiotropy analysis. (E-H) The reverse MR analysis for NADH on BRCA1. (E) The scatter plot of the association between NADH and BRCA1. (F) The forest plot was used to show the MR estimate and 95% CI value (black line segment) for each SNP. (G) The funnel plot of heterogeneity analysis. (H) The forest plot of pleiotropy analysis.

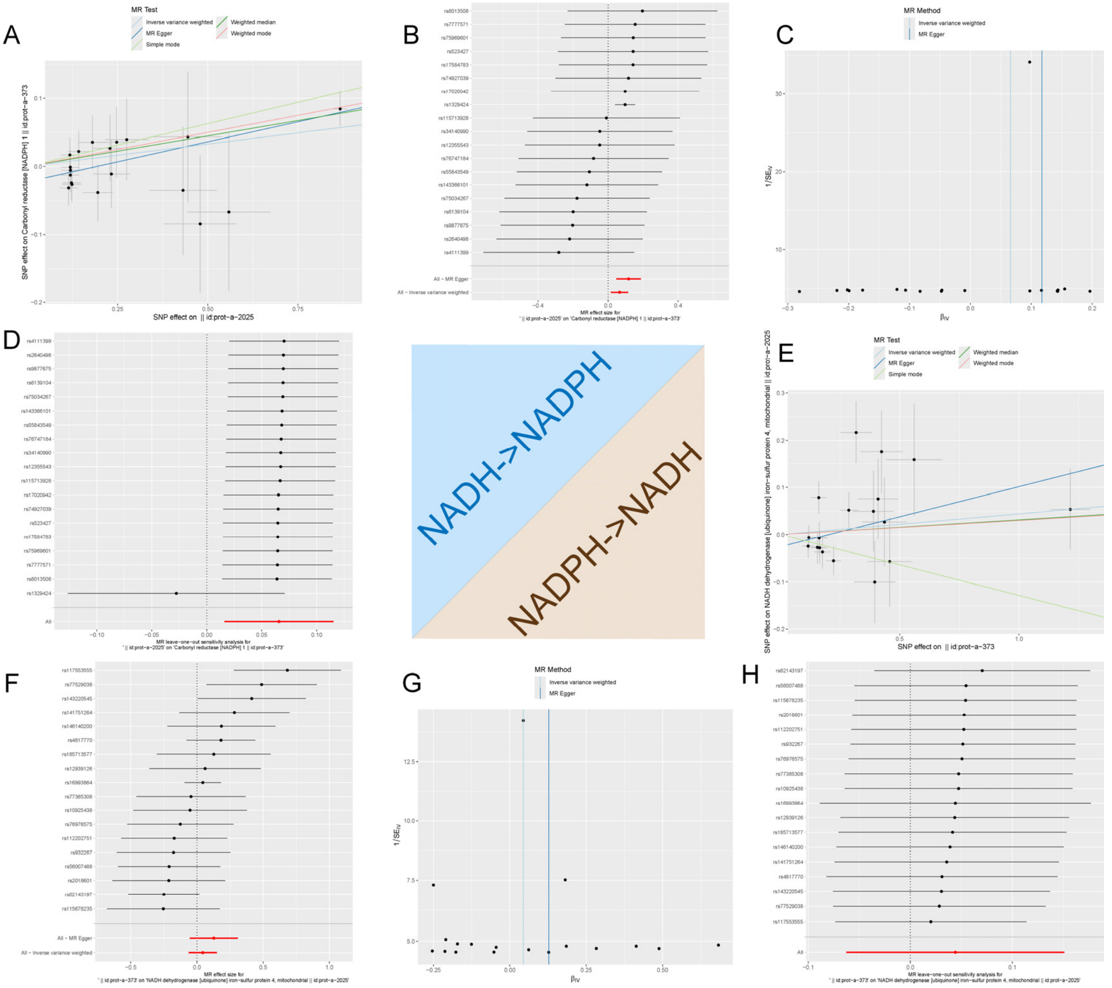

Supplementary Figure S7. (A-D)The forward MR analysis: casual effect of NADH on NADPH. (A) The scatter plot of the association between NADH and NADPH. (B) The forest plot was used to show the MR estimate and 95% CI value (black line segment) for each SNP. (C) The funnel plot of heterogeneity analysis. (D) The forest plot of pleiotropy analysis. (E-H) The reverse MR analysis for NADPH on NADH. (E) The scatter plot of the association between NADPH and NADH. (F) The forest plot was used to show the MR estimate and 95% CI value (black line segment) for each SNP. (G) The funnel plot of heterogeneity analysis. (H) The forest plot of pleiotropy analysis.

Supplementary Table S1. The data sources of the datasets used in this study.

**Datasets Name**	**Data Sources Links**	**Sources**
TCGA-STAD	https://portal.gdc.cancer.gov/	TCGA[30]
Clinical TCGA-STAD	https://portal.gdc.cancer.gov/	TCGA[30]
GSE13911	https://www.ncbi.nlm.nih.gov/geo/query/acc.cgi?acc=GSE13911	GEO[31]
GSE65801	https://www.ncbi.nlm.nih.gov/geo/query/acc.cgi?acc=GSE65801	GEO[32]
GSE163558	https://www.ncbi.nlm.nih.gov/geo/query/acc.cgi?acc= GSE163558	GEO[33]
GWAS-GC	https://opengwas.io/datasets/ebi-a-GCST90018629	GWAS[34]
GWAS-BRCA1	https://opengwas.io/datasets/prot-a-2004	GWAS[34]
GWAS-NADPH	https://opengwas.io/datasets/prot-a-373	GWAS[34]
GWAS-NADH	https://opengwas.io/datasets/prot-a-2025	GWAS[34]

Supplementary Table S2. The Filter Criteria of SNPs

Exposure	Outcome	P-Value	r^2	Number of retained SNPs
BRCA1	GC	$5*10^{-6}$	0.001	4
GC	BRCA1	$5*10^{-6}$	0.001	15
NADPH	GC	$5*10^{-6}$	0.001	8
GC	NADPH	$5*10^{-6}$	0.001	15
BRCA1	NADPH	$5*10^{-6}$	0.001	9
NADPH	BRCA1	$5*10^{-7}$	0.001	4
BRCA1	NADH	$5*10^{-6}$	0.001	9
NADH	BRCA1	$5*10^{-6}$	0.001	19
NADH	NADPH	$5*10^{-6}$	0.001	19
NADPH	NADH	$5*10^{-6}$	0.001	18

Supplementary Table S3. The Instrumental Variables

	BRCA1-GC	GC-BRCA1	NADPH-GC	GC-NADPH	BRCA1-NADPH	NADPH-BRCA1
1	rs148150385	rs1050437	rs10925438	rs1050437	rs11209435	rs16993864
2	rs2547245	rs111330133	rs146140200	rs111330133	rs115500871	rs4817770
3	rs34047734	rs2597736	rs2018601	rs2597736	rs148150385	rs56007488
4	rs62143197	rs2598405	rs4817770	rs2598405	rs2547245	rs62143197
5		rs3853601	rs56007488	rs3853601	rs34047734	
6		rs59585832	rs62143197	rs59585832	rs62143197	
7		rs72690905	rs77529038	rs72690905	rs77274666	
8		rs7542186	rs932267	rs7542186	rs78071024	
9		rs760077		rs760077	rs79546782	
10		rs7752906		rs7752906		
11		rs7849280		rs7849280		
12		rs8116385		rs8116385		
13		rs9273560		rs9273560		
14		rs9277947		rs9277947		
15		rs9405098		rs9405098		

Supplementary Table S4. The Instrumental Variables

	BRCA1-NADH	NADH-BRCA1	NADH-NADPH	NADPH-NADH	GC-NADH	NADH-GC
1	rs11209435	rs115713928	rs115713928	rs10925438	rs1050437	rs115713928
2	rs115500871	rs12355543	rs12355543	rs112202751	rs111330133	rs1329424
3	rs148150385	rs1329424	rs1329424	rs115678235	rs2597736	rs17584783
4	rs2547245	rs143366101	rs143366101	rs117553555	rs2598405	rs2640498
5	rs34047734	rs17020942	rs17020942	rs12939126	rs3853601	rs34140990
6	rs62143197	rs17584783	rs17584783	rs141751264	rs59585832	rs4111399
7	rs77274666	rs2640498	rs2640498	rs143220545	rs72690905	rs523427
8	rs78071024	rs34140990	rs34140990	rs146140200	rs7542186	rs55843549
9	rs79546782	rs4111399	rs4111399	rs16993864	rs760077	rs6139104
10		rs523427	rs523427	rs185713577	rs7752906	rs74927039
11		rs55843549	rs55843549	rs2018601	rs7849280	rs75034267
12		rs6139104	rs6139104	rs4817770	rs8116385	rs75969601
13		rs74927039	rs74927039	rs56007488	rs9273560	rs7777571
14		rs75034267	rs75034267	rs62143197	rs9277947	rs8013508
15		rs75969601	rs75969601	rs76976575	rs9405098	rs9877675
16		rs76747184	rs76747184	rs77385308		
17		rs7777571	rs7777571	rs77529038		
18		rs8013508	rs8013508	rs932267		
19		rs9877675	rs9877675			

Supplementary Table S5. Pleiotropy and heterogeneity analysis between BRCA1 and GC

Exposure	Outcome	No. of SNPs	MR-Egger regression		Heterogeneity analyses		
			intercept	p_intercept	Method	Q	Q_pval
BRCA1	GC	4	-0.017	0.923	MR-Egger	3.565	0.168
					IVW	3.586	0.310
GC	BRCA1	15	0.013	0.643	MR-Egger	23.372	0.037
					IVW	23.777	0.048

Supplementary Table S6. Pleiotropy and heterogeneity analyses between NADPH and GC

Exposure	Outcome	No. of SNPs	MR-Egger regression		Heterogeneity analyses		
			intercept	p_intercept	Method	Q	Q_pval
NADPH	GC	8	0.034	0.182	MR-Egger	4.606	0.595
					IVW	6.877	0.442
GC	NADPH	15	0.033	0.167	MR-Egger	14.578	0.334
					IVW	16.975	0.258

Supplementary Table S7. Pleiotropy and heterogeneity analysis between NADH and GC

Exposure	Outcome	No. of SNPs	MR-Egger regression		Heterogeneity analyses		
			intercept	p_intercept	Method	Q	Q_pval
NADH	GC	15	0.005	0.553	MR-Egger	5.945	0.948
					IVW	6.316	0.958
GC	NADH	15	-0.021	0.385	MR-Egger	16.332	0.232
					IVW	17.350	0.238

Supplementary Table S8. Pleiotropy and heterogeneity analyses between BRCA1 and NADPH

Exposure	Outcome	No. of SNPs	MR-Egger regression		Heterogeneity analyses		
			intercept	p_intercept	Method	Q	Q_pval
BRCA1	NADPH	9	-0.005	0.923	MR-Egger	9.826	0.199
					IVW	9.840	0.276
NADPH	BRCA1	4	0.181	0.373	MR-Egger	54.279	$1.63*10^{-12}$
					IVW	89.471	$2.85*10^{-19}$

Supplementary Table S9. Pleiotropy and heterogeneity analyses between BRCA1 and NADH

Exposure	Outcome	No. of SNPs	MR-Egger regression		Heterogeneity analyses		
			intercept	p_intercept	Method	Q	Q_pval
BRCA1	NADH	9	0.037	0.376	MR-Egger	6.513	0.481
					IVW	7.405	0.494
NADH	BRCA1	19	-0.01	0.46	MR-Egger	13.020	0.735
					IVW	13.602	0.755

Supplementary Table S10. Pleiotropy and heterogeneity analyses between NADH and NADPH

Exposure	Outcome	No. of SNPs	MR-Egger regression		Heterogeneity analyses		
			intercept	p_intercept	Method	Q	Q_pval
NADH	NADPH	19	-0.023	0.053	MR-Egger	9.250	0.932
					IVW	13.560	0.757
NADPH	NADH	18	-0.026	0.284	MR-Egger	30.434	0.016
					IVW	32.776	0.012
